# Near-zero-dispersion soliton and broadband modulational instability Kerr microcombs in anomalous dispersion

**DOI:** 10.1038/s41377-023-01076-8

**Published:** 2023-02-01

**Authors:** Zeyu Xiao, Tieying Li, Minglu Cai, Hongyi Zhang, Yi Huang, Chao Li, Baicheng Yao, Kan Wu, Jianping Chen

**Affiliations:** 1 State Key Laboratory of Advanced Optical Communication Systems and Networks, School of Electronic Information and Electrical Engineering, Department of Electronic Engineering, Shanghai Jiao Tong University, Shanghai, 200240 China; 2 Key Laboratory of Optical Fibre Sensing and Communications (Education Ministry of China), University of Electronic Science and Technology of China, Chengdu, 611731 China

**Keywords:** Solitons, Nonlinear optics

## Abstract

The developing advances of microresonator-based Kerr cavity solitons have enabled versatile applications ranging from communication, signal processing to high-precision measurements. Resonator dispersion is the key factor determining the Kerr comb dynamics. Near the zero group-velocity-dispersion (GVD) regime, low-noise and broadband microcomb sources are achievable, which is crucial to the application of the Kerr soliton. When the GVD is almost vanished, higher-order dispersion can significantly affect the Kerr comb dynamics. Although many studies have investigated the Kerr comb dynamics near the zero-dispersion regime in microresonator or fiber ring system, limited by dispersion profiles and dispersion perturbations, the near-zero-dispersion soliton structure pumped in the anomalous dispersion side is still elusive so far. Here, we theoretically and experimentally investigate the microcomb dynamics in fiber-based Fabry-Perot microresonator with ultra-small anomalous GVD. We obtain 2/3-octave-spaning microcombs with ~10 GHz spacing, >84 THz span, and >8400 comb lines in the modulational instability (MI) state, without any external nonlinear spectral broadening. Such widely-spanned MI combs are also able to enter the soliton state. Moreover, we report the first observation of anomalous-dispersion based near-zero-dispersion solitons, which exhibits a local repetition rate up to 8.6 THz, an individual pulse duration <100 fs, a span >32 THz and >3200 comb lines. These two distinct comb states have their own advantages. The broadband MI combs possess high conversion efficiency and wide existing range, while the near-zero-dispersion soliton exhibits relatively low phase noise and ultra-high local repetition rate. This work complements the dynamics of Kerr cavity soliton near the zero-dispersion regime, and may stimulate cross-disciplinary inspirations ranging from dispersion-controlled microresonators to broadband coherent comb devices.

## Introduction

Microresonators-based frequency combs have attracted intense interests in the last decade due to their revolutionary performances such as high coherence, flexible comb spacing, and broad bandwidth^1–9^. Diverse applications of microcombs including optical frequency synthesizer, atomic clock, lidar, spectroscopy, and optical communications have been reported^10–14^. In microresonators, chromatic dispersion is an essential physical quantity for determining the nonlinear dynamic process of the microcombs^15–18^. In the anomalous dispersion (AD) driven microresonator, microcombs can organize themselves to form a bright localized dissipative structure (LDS), also termed as dissipative Kerr solitons (DKSs)^19^, with maintained waveform determined by the “nonlinearity-dispersion” and “gain-loss” double balance^19^. In contrast, the microcombs in normal dispersion (ND) based microresonators permit the formation of dark LDS (also known as dark soliton), which are intimately related to the so-called switching waves (SWs)^20,21^. The SWs can be stabilized at their own Maxwell point, or interlocked with each other by oscillatory trailing^22^. This unique stabilization mechanism allows for enhanced comb conversion efficiency^23^.

Recently, numerous theoretical and experimental works focusing on the soliton dynamics in near-zero-dispersion driven microresonators have emerged. An obvious advantage of this scenario is that the small group velocity dispersion (GVD) enables a small spectral deviation of the comb from the nearby resonance, thus increasing the soliton spectral width. Near the zero-dispersion regime, since the GVD is almost vanished, the higher-order dispersion dominates the intracavity field evolution, thus significantly affects the soliton’s localized structure and stability dynamics. It has been theoretically predicted by the works of Parra-Rivas the perturbation induced by third-order dispersion (TOD) permits the existence of bright solitons in the normal dispersion regime^24,25^ and has been experimentally confirmed by the recent works in refs. ^26,27^. Also, the work of ref. ^26^ demonstrates the single-peak bright soliton can exist in the regime across the zero-dispersion wavelength. This bright soliton structure is based on the interlocking of up-SWs and down-SWs, and is termed as zero-dispersion soliton (ZDS) in ref. ^27^. As the soliton spectrum actually evolves in a wide spectral range across the zero-dispersion wavelength, we prefer to term it as near-zero-dispersion soliton (NZDS) in the following parts of this paper. Interestingly, it raises a natural question whether there exists a corresponding NZDS structure in the anomalous dispersion regime. Due to the presence of parametric gain provided by the modulational instability (MI) in the AD regime, the intracavity fields will experience completely different nonlinear dynamics compared to its counterpart in the ND regime. However, to date, no systematic study, especially experimental investigation, has been performed to verify this speculation.

In this work, by using a highly nonlinear fiber Fabry-Perot (F-P) microcavity, we explore the dynamics of Kerr comb generation in the near-zero anomalous-dispersion regime. We adopt a pulsed pumping scheme to effectively reduce the demand for average pumping power and alleviate the intracavity thermal effect^28–30^. Thanks to the ultra-small anomalous GVD, we have experimentally obtained a 2/3-octave-spaning microcomb in the broadband MI state with a spectrum from 1240 nm to 1950 nm and a mode spacing of 10 GHz. The corresponding number of comb lines is more than 8400. Moreover, we theoretically and experimentally reveal the generation of a novel soliton structure, which is composed of a series of bound solitons (soliton cluster). The soliton cluster has local repetition frequency up to 8.6 THz and individual pulse width is less than 100 fs in the time domain. The corresponding spectral span is >32 THz and the comb line number is >3200. These tightly packed multi-soliton structures are unambiguously identified through analysis of their spectral envelopes. Since these novel soliton structures exist in near-zero anomalous-dispersion regime where intracavity MI and third-order dispersion dominate the field evolution, we term them as “anomalous-dispersion based near-zero-dispersion soliton (AD-NZDS)”. The microcombs in broadband MI state and AD-NZDS state possess their own potential application scenarios. The MI microcomb state has advantages of high conversion efficiency and widely accessible range, which is suitable for the high power microcomb application. In contrast, the coherent spectrum of AD-NZDS state has relatively low phase-noise feature and self-organized structures, which provides unique capabilities in the applications of optical computing, light sensing, communication and spectroscopy, etc. This work provides a new sight into the nonlinear dynamics of the Kerr microcombs near the zero-dispersion regime and presents a flexible strategy to choose the operating state of the microcombs depending on the requirement of the application.

## Results

### Analysis of near-zero-dispersion microcomb evolution

**Fig. 1:**
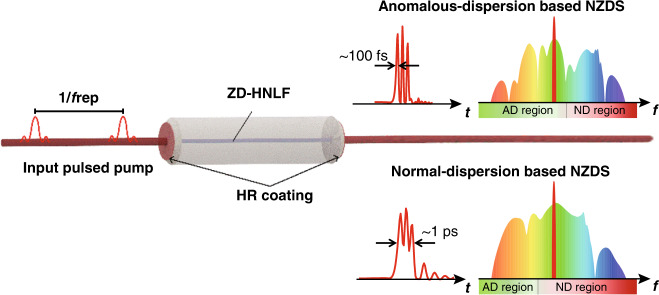
Conceptual illustration of NZDS generation in a pulse driven F-P microresonator. The upper and lower panels show the temporal waveforms and spectra of NZDSs pumped in the anomalous- and normal-dispersion sides respectively. ZD-HNLF: zero-dispersion highly nonlinear fiber, HR coating: highly reflective coating

Figure 1 illustrates the conceptual schematics of the generation of NZDS under a pulsed pump. Due to the presence or absence of intracavity MI effect, NZDSs obtained by pumping at near-zero anomalous- and normal-dispersion regimes have different localized-dissipative structures. The upper panel presents the generation of AD-NZDS, which is pumped in the near-zero anomalous-dispersion regime. Its temporal structures are composed of the interlocked pulses with ~100 fs duration. In contrast, the lower panel shows the generation of normal-dispersion based NZDS (ND-NZDS), which is pumped in the near-zero normal-dispersion regime. It has multi-peak profiles in a single pulse with ~1 ps duration made up by the interlocking of up- and down-switching waves, which has been extensively investigated in the works of refs. ^24–27^. As our work is focused on the investigation of AD-NZDS, the term “NZDS” in the following refers to the AD-NZDS structure studied in this paper.

**Fig. 2:**
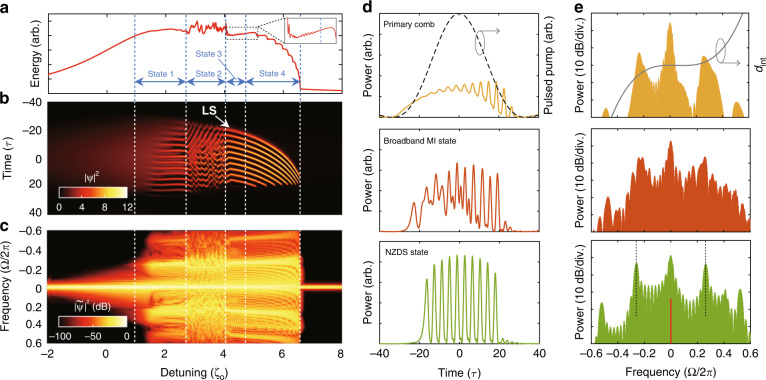
Simulation of anomalous-dispersion based near-zero-dispersion soliton via desynchronized pulse pumping. **a** Intracavity power, **b** temporal and **c** spectral evolution of near-zero-dispersion soliton with increased detuning. States 1 to 4 correspond to the primary comb, broadband MI, unstable NZDS and NZDS states. Inset in **a** shows a zoomed view of unstable NZDS power fluctuation. The white arrow in **b** marks the position of leading soliton (LS). **d** Temporal waveforms and **e** spectral profiles of primary comb state (*ζ*_0_ = 1.6), broadband MI state (*ζ*_0_ = 3.2), and NZDS state (*ζ*_0_ = 4.8). In the NZDS state, the black-dashed lines mark the position of two prominent combs near the pump denoted by the red line. The gray dashed line in (**d**) plots the pulsed pump envelope. The gray solid line in (**e**) depicts the normalized *d*_int_ curve for *d*_2_ = −1, *d*_3_ = 1

To investigate the universal microcomb evolution in the microresonator that pumped in the near-zero anomalous-dispersion regime, we perform the numerical simulation in the normalized Lugiato-Lefever Equation for Fabry-Pérot cavity (FP-LLE) (see “Materials and Methods”)^31,32^. In the simulation, the F-P microresonator is driven at anomalous-dispersion regime and possesses relatively strong TOD (considering GVD parameter *d*_2_ = −1, TOD parameter *d*_3_ = 1). The normalized *d*_int_ profiles see gray line in Fig. 2e, where *d*_int_ = *d*_2_Ω^2^ + *d*_3_Ω^3^ (Ω is the normalized angular frequency, and the details of all parameter normalization can be referred to “Materials and Methods”). Under the influence of TOD, it breaks the symmetry in the temporal and spectral profiles, which leads to a constant-velocity temporal drift^33^. To compensate this group-velocity shift, the drift coefficient *d* is set as −2.4, which introduces a proper counter-acting group-velocity shift and helps to maintain the soliton structure. The drift coefficient *d* is defined as the normalized group-velocity difference between the intracavity fields and the pulsed pump (details in “Materials and Methods”). The pulsed pump is composed of total *M* + 1 = 51 spectral lines with equal phase and power, and its normalized peak power *F* is set as 12. The waveform profile of the driving pulse is plotted in the gray curve in Fig. 2d. The detuning is scanned linearly from *ζ*_0_ = −2 to *ζ*_0_ = 8, which allows the intracavity fields to experience all possible states. The intracavity field evolutions in time domain and frequency domain are shown in Fig. 2b and c, and the corresponding power variation is exhibited in Fig. 2a. During the laser scanning, four typical optical states are identified, and their region are marked by the dashed lines in Fig. 2a–c. Figure 2d and e presents the typical temporal waveforms and spectral profiles in primary comb state, broadband MI state, and NZDS state with the detuning *ζ*_0_ = 1.6, 3.2, and 4.8, respectively. When the detuning is swept to *ζ*_0_ = 1.6, the MI allows the power conversion from the driving modes to the primary comb envelopes (yellow line in Fig. 2e), which will modulate the temporal waveform of intracavity driving pulse and generate a series of little peaks on its top (yellow line in Fig. 2d). As the detuning is swept to *ζ*_0_ = 3.2, the further build-up of intracavity power leads to the instability of the temporal structure, and the fields evolve to the broadband MI state (orange line in Fig. 2d). In this state, broadband microcombs can be generated on the spectrum (orange line in Fig. 2e). When the detuning *ζ*_0_ exceeds 4, the intracavity field enters the unstable NZDS state. In the unstable NZDS state, some of the pulses in the soliton cluster enter the stable state, while some others do not, which can be identified in the temporal evolution in Fig. 2b. Such unstable NZDS forms a jitter soliton step feature on the transmission curve in Fig. 2a (see inset). When the detuning *ζ*_0_ increases further, the stable multi-soliton binding structure (soliton cluster) appears in temporal evolution (Fig. 2b). The temporal waveform at *ζ*_0_ = 4.8 is presented by green line in Fig. 2d, which consists of 10 bound solitons with equal time intervals. Such tightly packed soliton structures lead to coherent spectral envelopes in the frequency domain, as shown in green line of Fig. 2e. With further increase of the detuning, the number of bound solitons decreases one by one until a 2-binding soliton state is obtained at *ζ*_0_ = 6.5. The mechanism of continuous decrease in pulse number has been discussed in Supplementary Section 2.

These novel multi-soliton states observed in the simulations originate from the intracavity MI and the TOD effects. We term this near-zero-dispersion soliton state as NZDS^(n)^ based on the number of bound solitons. The orders of NZDS^(n)^ can be also identified by the number of coherent envelopes between the prominent combs and pump envelopes in the frequency domain. The prominent combs refer to the high-power comb envelopes in the spectrum of the NZDS state, as marked by the black-dashed lines in Fig. 2e. In the case of proper resonator dispersion, the higher-order NZDS^(n)^ state can continuously evolve into NZDS^(1)^ state, that exhibits a structure of conventional DKS with dispersive-wave tails (see Supplementary Section 5).

### Observation of stable near-zero-dispersion soliton

**Fig. 3:**
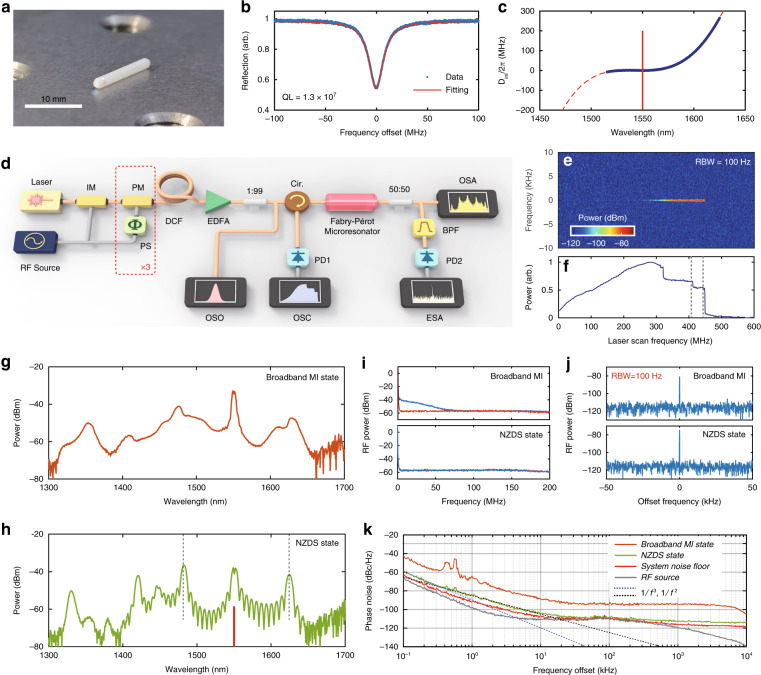
Experimental AD-NZDS formation via desynchronized pulse driving. **a** Photograph of the F-P microresonator. **b** Reflective spectrum of the microresonator when the C.W. laser is scan over a resonance near 1550 nm. Blue dots: measured data. Red curve: Lorenzian fit. **c** Measured integrated dispersion *D*_int_ (blue dots) and fitting curve (red dashed line). The center mode of the driving pulse is indicated by a red solid line. **d** Experimental setup. IM: intensity modulator, PM: phase modulator, EDFA: erbium-doped fiber amplifier, PS: phase shifter, ESA: electronic spectrum analyzer, DCF: dispersion compensation fiber, Cir: circulator, PD: photodetector, OSA: optical spectrum analyzer, OSO: optical sampling oscilloscope, OSC: oscilloscope. **e** Evolution of the repetition-rate beatnote and **f** resonator transmission versus various laser scan frequency. The gray-dashed lines mark the measured detuning positions in (**g**) and (**h**). Spectral profiles of **g** broadband MI state, and **h** NZDS state. The black-dashed lines mark the position of prominent combs, and red-solid line marks the position of pump envelope. **i** Intensity noise (blue) in the broadband MI and NZDS states. The ESA noise floor is in red curves. **j** Repetition-rate beatnote signal in the broadband MI and NZDS state. **k** Phase noise spectra of broadband MI state (orange) and NZDS state (green), along with the phase noise of RF source (gray) and system noise floor (red)

In our experiment, we investigate the near-zero-dispersion soliton dynamics in a highly nonlinear fiber based Fabry-Pérot microresonator. The photograph of the F-P microresonator is presented in Fig. 3a. It has a cavity length of ~1 cm, and a free spectral range (FSR) of 10.41506 GHz which is precisely measured by a dual-cavity based measurement (see Supplementary Section 6). The reflection signal across the resonance near 1550 nm is presented in Fig. 3b, exhibiting a resonance width of 15 MHz that corresponds to a loaded *Q* of 1.3 × 10^7^. The details on the parameters and fabrication of the fiber F-P microresonator are provided in “Materials and Methods”. Compared with other microcavity platforms, the fiber F-P microresonator has the advantages of easy coupling, low intracavity loss, and mature dispersion engineering^34,35^, which is ideal for near-zero-dispersion soliton research. Figure 3c exhibits the integrated dispersion profile of the microresonator, which is measured by fiber Mach-Zehnder interferometer method (see Supplementary Section 6)^36^. The measured *D*_int_ is fitted by a polynomial centered at 1550 nm, and has *D*_2_/2π = 167 Hz, and *D*_3_/2π = −1.9 Hz, which exhibits very small anomalous GVD and small TOD. In dimensionless expression, we have the value of *d*_2_ = −1, and *d*_3_ = 1.14. The experimental setup is shown in Fig. 3d. The F-P microresonator is driven by a pulse pumping scheme, and its repetition rate can be precisely controlled by the loaded radio frequency (RF) signal. The generated pulsed pump has 49 electro-optic comb lines in −10 dB range, and the corresponding pulse duration is measured to be 2.1 ps. Details of the experimental setup and procedure can be found in “Materials and Methods”.

The desynchronization frequency of the driving pulse is defined as *δf*_rep_ = *f*_rep_ – *D*_1_/2π. Such desynchronization frequency can introduce a counter-acting effect against the constant-velocity temporal drift caused by intracavity TOD, and helps to maintain the soliton structure^27^. In the experiment, we set desynchronization *δf*_rep_ = –20 kHz to provide a proper group-velocity compensation. The central wavelength of the driving pulse is slowly tuned across the resonance at 1549.55 nm from blue-detuning to the red-detuning region with a scanning speed of 20 MHz∙s^-1^and an average coupling-in power of 23.5 dBm. The F-P microresonator is driven in the anomalous-dispersion regime. The output light of the F-P microresonator is continuously measured by an optical spectrum analyzer (OSA) and an electronic spectrum analyzer (ESA). A bandpass filter with a central wavelength of 1580 nm and bandwidth of 0.02 nm is used for beatnote measurement. Figure 3e exhibits the obtained RF repetition-rate beatnote signal of the output spectrum, and the corresponding power variation is recorded in Fig. 3f. The frequency coordinates in Fig. 3e and f represent the change of pump laser frequency rather than the pump-resonance detuning. When laser wavelength is swept to the “step” feature in Fig. 3f, the beatnote signal is obviously enhanced. The spectral profiles at the end of each “step” are shown in Fig. 3g and h, respectively. When the intracavity field is at MI state, broadband Kerr microcomb is observed, as shown in Fig. 3g. The obtained spectrum is characterized by obvious wave peaks, which originate from the residual primary comb as well as the transmission spectrum of the dielectric mirror coating. The MI microcomb has broadband intensity noise, as shown in the top panel of Fig. 3i. The existing region of broadband MI state is much larger than that of the soliton state, as a result of negative feedback provided by the thermo-optic effect^19^. Since the power variation in Fig. 3f is measured by a low-sampling-rate power meter, noise jitter is not observed on the broadband MI region and the unstable NZDS region. When the broadband MI spectrum evolves into a spectrally coherent NZDS state (Fig. 3h), the comb intensity noise has dropped below the noise floor of the ESA (bottom panel of Fig. 3i), which corresponds to a highly coherent mode-locked state. Consistent with numerical simulation, the spectra of NZDS are characterized by a collection of coherent envelopes. The prominent combs (marked by dashed lines in Fig. 3h) and pump envelope are connected by a number of minor coherent envelopes. According to the numbers of minor envelopes, we can identify the obtained NZDS states as NZDS^(11)^, whose temporal structures are composed of 11 bound solitons within the time window of the driving pump pulse.

To further characterize the comb noise of the broadband MI and NZDS states, we measure their repetition-rate beatnote signal and single-sideband phase noise (SSB-PN) at 1580 nm, as shown in Fig. 3j and k. When the intracavity field is at the broadband MI state, it exhibits an extinction ratio of 35.9 dB, and a central frequency of 10.41504 GHz, which is precisely equal to the repetition rate of the driving pulse. When the intracavity field evolves to the NZDS^(11)^ state, it exhibits an enhanced extinction ratio of 42.2 dB. As shown in Fig. 3k, the phase noise of the NZDS^(11)^ state is ~20 dB lower than that of the broadband MI state, which exhibits <104 dBc Hz^−1^ at 10 kHz, and <114 dBc Hz^−1^ at 10 MHz. The NZDS state demonstrates relatively low phase noise, which follows the noise floor of the system. Such phase noise is comparable to that of Kerr soliton in the pure anomalous GVD regime^18^. At low frequency (100 300 Hz), the NZDS^(11)^ comb noise follows a 1/*f* ^3^ fitting in the SSB-PN spectrum, which depends on the flicker frequency noise from the RF source^35^. From 300 Hz to 10 kHz, the NZDS^(11)^ comb noise shows a 1/*f* ^2^ dependence, which is limited by the white noise from the RF source.

### Broadband MI microcomb states

**Fig. 4:**
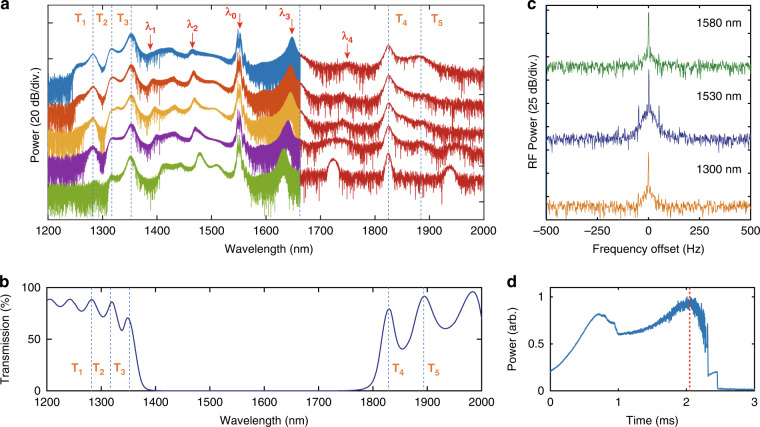
MI microcombs with 2/3-octave spanning. **a** Broadband MI microcomb spectra. The corresponding pump power from the bottom (green) to the top (blue) of the spectra are 23.5 dBm, 24.5 dBm, 25 dBm, 25.5 dBm, and 26 dBm. Four residual primary comb envelopes are marked by *λ*_1_–*λ*_4_, and the mirror coating induced fixed peaks are marked by T_1_–T_5_. **b** Measured transmission spectrum of dielectric mirror coating. The transmission peaks are also marked by T_1_–T_5_. **c** Repetition-rate beatnote signals of MI microcomb near 1300 nm (yellow), 1530 nm (blue), and 1580 nm (green). **d** Transmission curve for *δf*_rep_ = –20 kHz and pump power of 26 dBm. The red dashed line marks the measured position of the broadband MI spectrum as shown in blue curve in (**a**)

With the scanning of pump frequency, the primary comb first evolves to a broadband MI microcomb state. With our near-zero-dispersion fiber F-P microresonator, we experimentally obtained a widely accessible broadband MI state with 2/3 octave spanning thanks to the ultra-small cavity GVD. Figure 4a summarizes the widest MI microcomb spectra that can be obtained under different coupling-in pump power ranging from 23.5 dBm, 24.5 dBm, 25 dBm, 25.5 dBm, to 26 dBm, respectively (from bottom to top). The spectra are measured by two optical spectrum analyzers, with one measuring the range of 1200–1660 nm and the other of 1660–2000 nm. The measured transmission spectrum of the dielectric mirror coating of the F-P microresonator is presented in Fig. 4b for comparison, whose transmission peaks are marked by T_1_–T_5_. In the high reflection region of mirror coating (1360−1760 nm), four residual primary comb envelopes (marked by *λ*_1_−*λ*_4_) are identified in Fig. 4a. With the increase of pump power, these primary comb envelopes shift away from the central driving mode (marked by *λ*_0_), which is consistent with the theoretical predictions^19,37^, while the peaks (marked by T_1_–T_5_) generated by the transmission peaks of dielectric mirror coating remain fixed. Benefiting from the leakage of the intracavity optical field, we can obtain an ultra-wide spectrum in the broadband MI state. At the injected pump power of 26 dBm (blue curve in Fig. 4a), the measured comb spectrum covers from 1240 nm to 1950 nm (more than 2/3 octave in −40 dB range) with 10-GHz comb line spacing, which contains more than 8400 comb lines. The corresponding output power of the MI microcomb is ~−17 dBm estimated from the spectrum. Figure 4c presents the repetition-rate beatnote signal near 1300 nm, 1530 nm, and 1580 nm. Their beatnote signals show an extinction ratio larger than 35 dB. In ref. ^5^, it demonstrates a chaos-assisted two-octave-spanning microcombs with FSR of 0.9 THz, which contains ~1000 comb lines. In our work, benefited from the ultra-small anomalous GVD, we obtained a much larger number of comb lines than Ref. ^5^. In our experiments, the bandwidth of broadband MI comb is mainly limited by the reflection bandwidth of the mirror coating. The further broadening of the comb spectrum can be achieved by superimposing the mirror coating at different central wavelengths. When the laser scanning speed increases to 80 GHz s^−1^, the soliton formation step can be observed after the unstable MI comb steps, as shown in Fig. 4d. It is indicated that this broadband MI microcomb state can also enter the mode-locked soliton state. We will discuss the temporal structure of the soliton state in the next section.

Although the MI microcomb reveals a relatively poor noise feature, it offers an easily accessible broadband multi-wavelength source with high conversion efficiency, which has promising application in optical coherence tomography, optical cryptography systems and Lidar. A long-term stability experiment in the MI microcomb state has been demonstrated in Supplementary Section 8 only by a simple feedback method.

### NZDS dynamics versus different desynchronization frequency

**Fig. 5:**
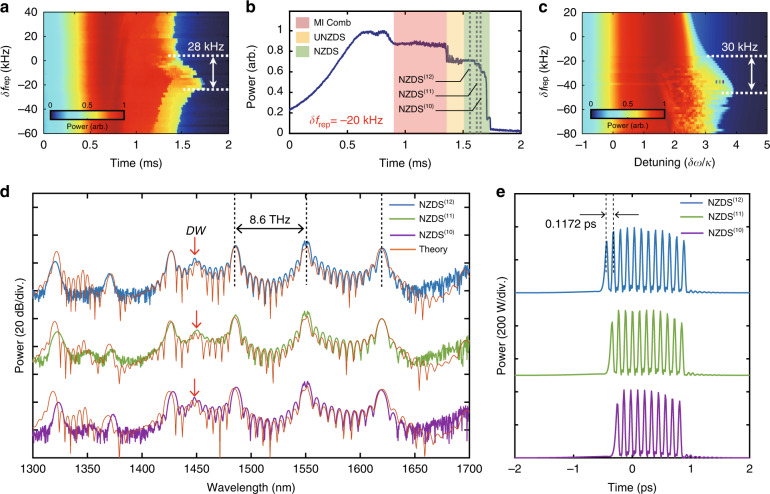
Observation of tightly bound temporal structures in NZDS(10-12) states. **a** Contour plot of the resonator transmission at different desynchronization value of *δf*_rep_. **b** Transmission curve for *δf*_rep_ = –20 kHz. The broadband MI state, unstable NZDS (UNZDS) state and NZDS state are indicated by the red, yellow and green areas, respectively. Gray dashed lines mark the different step positions of the NZDS state demonstrated in **d**. **c** Simulated NZDS step profiles as a function of detuning and desynchronization frequency. The color map represents intracavity normalized power. **d** Experimentally obtained microcomb spectra at different NZDS states, along with the spectrum simulation results (orange curve). These three states are also marked by blue, green and purple points in (**c**). The dispersive waves are marked by red arrows in (**d**). The corresponding temporal simulation results are presented in (**e**)

For the generation of NZDS with pulsed pump, the pump repetition rate *f*_rep_ is not required to be strictly equal to the FSR of the resonator, and the generated Kerr solitons can be locked with the driving pump pulse at a certain desynchronization^29^. Particularly, when TOD dominates the intracavity field, it leads to a constant-velocity temporal drift, and a proper desynchronization of the driving pulse helps to offset this temporal drift which can extend the Kerr soliton existing range^27,33,38^. In this section, we explore the existing range of NZDSs at different desynchronization frequency *δf*_rep_. The relation between the maximum pulse number and their position versus desynchronization *δf*_rep_ has been discussed in detail in Supplementary Section 4. To determine the potential existence range of NZDSs, we repeatedly scan the central driving mode of the pulsed pump over the resonance at 1549.55 nm with an input average pump power of 23.5 dBm and a fast scanning speed of 80 GHz s^−1^, which helps to mitigate intracavitary thermal effects. Figure 5a exhibits the evolution of transmission spectra at different desynchronization values of *δf*_rep_. The region where NZDSs can be obtained ranges from *δf*_rep_ = –25 kHz to *δf*_rep_ = 3 kHz, for a total of 28 kHz. The transmission spectrum for *δf*_rep_ = –20 kHz is shown in Fig. 5b. In its red detuned region, a set of discrete steps can be observed, which corresponds to different NZDS states. The simulated NZDS step profiles as a function of desynchronization frequency *δf*_rep_ are shown in Fig. 5c, with the color map indicating intracavity normalized power. The numerical simulation is performed in a full-system FP-LLE model containing Raman effect and wavelength-dependent coupling loss of the dielectric mirror coating (see Materials and Methods). We use modified dispersion parameters of *D*_2_/2π = 40.6 Hz, and *D*_3_/2π = −0.7 Hz, corresponding to a normalized dispersion of *d*_3_/*d*_2_ = 5. From Fig. 5c, it demonstrates that the unfolding soliton steps range from *δf*_rep_ = –46 kHz to *δf*_rep_ = –16 kHz, for a total of 30 kHz. The simulated results shown in Fig. 5c are qualitatively consistent with experimental results in Fig. 5a. However, the soliton step profiles in the simulation are more biased towards the lower *δf*_rep_. We attribute the discrepancy to the intracavity temperature variation between resonator FSR measurement and the desynchronization experiment, which results in different FSR values and desynchronization frequency under distinct measurement conditions. Figure 5d shows the experimentally obtained optical spectra of NZDS^(12)^, NZDS^(11)^, and NZDS^(10)^, whose step locations are indicated by dashed lines in Fig. 5b. These three NZDS states are obtained by manually stabilizing the laser wavelength at the soliton step position. Although, some lower soliton steps are observed in Fig. 5b, these lower-order soliton states are experimentally thermal inaccessible due to the intracavity thermal effects^39^. To generate the lower-order NZDS^(n)^, we adopt an auxiliary laser pump scheme to mitigate the thermal effect, and successfully obtain the NZDS^(2)^ and NZDS^(3)^ in our experiment (details see Supplementary Section 7).

The orange curves in Fig. 5d show the corresponding numerical simulation results from full system FP-LLE model with experimental parameters, and the specific location of these solutions are marked by blue, green and purple dots in Fig. 5c, which corresponds to the detuning *δω*/*κ* = 3.2, 3.32, and 3.42, respectively. Due to the measurement difference in FSR values, we select desynchronization frequency *δf*_rep_ = –38 kHz in the simulation. The detailed simulation results of the optical field evolution are shown in Supplementary Section 3. The experimentally obtained and numerically simulated spectra in Fig. 5d show very good agreement. The only noticeable difference is that the comb envelope near 1350 nm. We assume that is related to the deviation of the transmittance data utilized in the model, and the comb lines near 1350 nm are submerged in the OSA noise floor. Our simulation further demonstrates the corresponding temporal structures in Fig. 5e, which are made up of a series of interconnecting individual soliton structures. The number of bound solitons provides a noticeable feature for distinguishing different NZDS states. From our experimental and simulated results, the proposed NZDS structure has the advantage of producing dense soliton cluster. For example, in the NZDS^(12)^ state, a local repetition frequency up to 8.6 THz and an individual pulse duration less than 100 fs can be achieved. This unique temporal structure will extend the application potential of Kerr soliton in optical computation, signal processing, and sensing.

## Discussion

From the theoretical model and simulation, the leftmost soliton of NZDS in the time domain (e.g., Fig. 5e) is substantially different in shape from the other bound solitons, and we term this soliton as the leading soliton marked by white arrow in Fig. 2b. During the optical field evolution, the leading soliton will be stabilized first, and intracavity MI perturbation and TOD-induced dispersive wave generate noisy background after the leading soliton. With the increasing of pump-resonance detuning, a discrete set of dissipative solitons can be evolved from the noisy background, and form the binding structure of NZDS. The spectrum of this bound soliton cluster is characterized by the prominent comb envelopes, and their frequency locations are consistent with that of the primary combs, as shown in Fig. 2c. Moreover, in the Supplementary GIF for Fig. S1, we demonstrate the evolution process from the conventional single soliton to the proposed near-zero-dispersion soliton in the *d*_2_/*d*_3_ coordinate. It can be seen that the leading soliton with dispersive wave tails will appear first and the newly generated soliton evolves from these oscillatory tails. Since the group velocity of the oscillatory tails and solitons are different, the temporal interval of the newly generated solitons automatically adjusts under the effect of nonlinearity and intracavity dispersion, and finally forms a bound soliton cluster. For the sake of easy discussion, we only demonstrate the field evolution of NZDS in the pulse pumping case in the theory section. The theory of NZDS is also applicable in the CW pumping scenario (see Supplementary Section 1).

Figure 5d presents the optical spectral profiles of NZDS^(12-10)^. Although, these spectra are in different soliton states, they share some similar features. First, a distinct dispersive wave around 1450 nm is observed in each NZDS spectra (marked by red arrow), and its position can be predicted by the dispersive wave theory^40^ (see Supplementary Section 7). Second, the NZDS spectra are all characterized by prominent comb envelopes, arising from the spectral evolution of the primary combs. The frequency interval between the prominent comb and pump envelopes determines the time interval between the bound solitons. For example, in the NZDS^(12)^ state (Fig. 5d), the frequency spacing of the comb envelope is 8.6 THz, and 1/8.6 THz ≈ 0.1172 ps is approximately the temporal separation between each discrete soliton. Since, the prominent combs evolve from the primary combs, the maximum frequency spacing between the prominent combs and pump envelope can be approximated by the position of the first generated primary comb. We assumed that the external driving power is equal to the primary comb threshold, the relative mode number of the first sidebands are given by $\mu _{th} = \sqrt {\kappa /D_2}$, where the *κ* is resonator decay rate, and *D*_2_ is second-order dispersion^19^. Using the simulation parameter given in the Materials and Methods, we set *κ*/2π = 30 MHz, and *D*_2_/2π = 40.6 Hz. Thus, we obtain that *μ*_th_ = 860, corresponding to the frequency spacing of 8.9 THz.

Next, we discuss the differences in Kerr comb dynamics that are pumped at different sides of the zero-dispersion wavelength. In the near-zero anomalous-dispersion regime, intracavity field will experience the MI state, since the resonator still inherently satisfies the phase-matching condition of MI. With the increase of pump-resonance detuning, the intracavity field gradually stabilizes into the AD-NZDS state, and the number of pulses in the soliton cluster decreases one after another until the equilibrium of the NZDS state is broken. In comparison, due to the lack of MI effect in the near-zero normal-dispersion regime, intracavity field will first enter the switching wave state during the scan of the pump laser across the resonance^27^. And then, with the further increase of detuning, the up- and down- SWs bond with each other to form the stable ND-NZDS, whose soliton orders also gradually decrease one by one, as shown in the experiment in ref. ^27^.

In addition, we have demonstrated that the fiber F-P microresonator is very suitable to investigate the Kerr microcomb dynamics near the zero-dispersion regime. First, compared to the integrated multimode microresonators, the fiber F-P microresonators have no complex avoided mode-crossing effects. The use of single-mode fiber allows only two orthogonal modes to be transmitted in the cavity. Second, compared to fiber ring resonators with long cavity length, the structure of fiber F-P microresonators is very simple and avoids the periodic perturbations caused by fiber couplers and dispersion-managed fiber. Third, a wide range of commercial fibers are available, making it easy to select the required dispersion design. The use of a widely tunable external-cavity diode laser could further increase the selection freedom of d_2_/d_3_ parameter.

Finally, we demonstrate that the investigation of microcomb near the zero-dispersion regime can provide a flexible strategy to generate microcombs based on the application requirements. The MI state is one of the most widely accessible microcomb states, and has broadband spectral profiles as well as high power conversion efficiency^41^. Our experiment has achieved the MI microcombs with more than 2/3-octave span, 10 GHz spacing and over 8400 comb lines (−40 dB) without any external nonlinear spectral broadening. Through numerical simulation methods, the average power conversion efficiency in the broadband MI state is 8.7% (Supplementary Section 3). Moreover, the broadband MI microcombs can continuously operate for more than 15 h through simple feedback controls, as shown in Supplementary Section 8. The MI microcombs are suitable for the application requiring broadband spectral coverage with dense comb lines and without much concern for noise characteristics. In contrast, the NZDS state has high spectral coherence and much lower phase noise. Although the spectral width of NZDS state is narrower than that of MI microcomb state, its bandwidth still covers more than 250 nm (−40 dB), which contains more than 3200 mode-locked comb lines. The temporal waveform of the NZDS state consists of a series of interlocked femtosecond pulses, i.e., soliton cluster. This closely arranged soliton cluster can be useful for the applications of optical computing and light sensing. The comparison between MI state and NZDS state is summarized in Table 1. In summary, this study extends the understanding of the Kerr cavity soliton dynamics near the zero-dispersion regime, and provides a new paradigm to generate broadband multi-wavelength optical sources with dense comb lines.

**Table 1:** The comparison of broadband MI state and NZDS state

	Broadband MI state	NZDS state
Spectral coverage	1240 nm to 1950 nm (>8400 comb-lines)	1400 nm to 1650 nm (>3200 comb-lines)
Advantages	1. High conversion efficiency; (Average efficiency of 8.7%); 2. Widely existing range.	1. Low phase noise; 2. Ultra-high local repetition rate.
Disadvantages	High phase noise.	1. Low conversion efficiency; (Efficiency from 3.8% to 5.7%); 2. Coherent spectrum with poor flatness
Applications	1. Lidar; 2. Spectrum communication; 3. Optical cryptography; 4. Optical coherence tomography.	1. Optical computation; 2. Light sensing; 3. Spectroscopy.

We would like to acknowledge a parallel work by Zhang et al.^54^ during the preparation of our manuscript. This work observes similar AD-NZDS and ND-NZDS structures in a fused silica microtoroid with CW pumping.

## Materials and methods

### Normalized FP-LLE model

To analyze the universal trends in dynamics and characterization of the near-zero-dispersion soliton, we use normalized Lugiato-Lefever equation for Fabry-Perot resonator (FP-LLE) with a pulse driving term *S*(*τ*)^31,32^.

1 $$\begin{array}{l}\frac{{\partial \psi }}{{\partial t^{\prime} }} = \left( { - d\frac{\partial }{{\partial \tau }} - id_2\frac{{\partial ^2}}{{\partial \tau ^2}} + d_3\frac{{\partial ^3}}{{\partial \tau ^3}}} \right)\psi \\ \qquad \quad+ \left( {i\left| \psi \right|^2 + 2i\left\langle {\left| \psi \right|^2} \right\rangle - i\zeta _0 - 1} \right)\psi + S(\tau )\end{array}$$

Here, *ψ* is the intracavity field envelope, *t*' is the normalized slow time, *τ* is the normalized fast time, and *ζ*_0_ is the normalized detuning of the central driving mode from the closest cavity resonance. The drift coefficient *d* describes the group-velocity difference between the field envelope *ψ* and the pulse-driving term *S*(*τ*). The parameters of *d*_2_ and *d*_3_ indicate the normalized second-order and third-order dispersion. In the normalized simulation, we set *d*_2_ = −1 for the scenario of anomalous GVD, thus *d*_3_ describes the strength of TOD relative to *d*_2_. The nonlinear term 2*i*〈|*ψ*|^2^〉*ψ* indicates the additional phase shift introduced in Fabry-Perot cavity^29,32^, where symbols 〈∙〉 denotes the field average over time domain, and has

2 $$\langle {\left| \psi \right|^2} \rangle = \frac{1}{{\tau _R}}\mathop {\int}\nolimits_{\tau _R/2}^{\tau _R/2} \left| \psi \right|^2d\tau$$

where *τ*_*R*_ is the normalized roundtrip time. The pulse-driving pump *S*(*τ*) is composed of *M* + 1 spectral comb lines with equal phase and power, and defined as

3 $${{{\mathrm{S}}}}\left( {{{\mathrm{\tau }}}} \right) = \frac{{\sqrt F }}{{M + 1}}\mathop {\sum }\limits_{\mu = - M/2}^{\mu = M/2} e^{i2\pi \mu f_{rep}\tau }$$

where *F* and *f*_rep_ represent the normalized peak power and repetition rate of the driving pulse, respectively. The details about parameter normalization can be referred to next section.

### Full system FP-LLE model

Experimental NZDS results are described by the full-system FP-LLE model with real parameters^32,42–45^. Considering the ultra-wide spectrum of NZDS, we include the Raman response and frequency-dependent intracavity loss in the FP-LLE^46,47^. The modified FP-LLE in frequency domain can be expressed as

4 $$\begin{array}{l}\frac{{\partial \tilde A\left( {{t},{\mu}} \right)}}{{{\partial} {t}}} = - \,{i}\left({D}{\mu} + \frac{{{D}_{2}}}{2}{\mu} ^2 + \frac{{{D}_{3}}}{6}{\mu} ^{3} + {\delta} {\omega} \right){\tilde {A}}- \frac{{{\kappa} \left( {\mu} \right)}}{2}\tilde {A}\\ \qquad\qquad + \,\sqrt {{\kappa} _{ext}\left( {\mu} \right)} {F}_{\mu} {(t)}+ \,{ig}_{0}{{{\mathcal{F}}}}\left[\right. {\left( {{1} - {f}_{R}} \right)\left| {A} \right|^{2A}}\\ \qquad\qquad + \,{{{f}_{R}}( {{h}_{R}( {T} ) \otimes \left| {A} \right|^{2}} ){A} + {2}\langle {\left| {A} \right|^{2}} \rangle {A}} \left]\right. \end{array}$$

where $\tilde A$(*t*, *μ*) represents the photon numbers in mode index *μ* with slow-time frame *t*. In the time domain, *A*(*t*, *T*) indicates intracavity photon flux, related to fast-time frame *T*, and has the following expression^27^.

5 $$A\left( {t,T} \right) = \mathop {\sum }\limits_\mu \tilde A_\mu (t)e^{ - i\mu D_1T}$$

The drift coefficient *D* = 2π (*f*_*re*p_ − *D*_1_/2π) indicates the angular desynchronization frequency between the driving pulse repetition rate and the cavity FSR^27,48,49^. The parameters of *D*_2_ and *D*_3_ represent the second and third-order dispersion. *δω* is the central driving mode detuning with respect to the nearby resonance. The spectrum-dependent cavity decay rate *κ* (*μ*) = *κ*_0_ + *κ*_*ext*_ (*μ*) with the intrinsic decay rate *κ*_0_ = 2π × 15 MHz and coupling decay rate *κ*_*ext*_ (*μ*) is determined by the reflection spectrum of mirror coating and *κ*_*ext*_ (*μ* = 0) = 2π × 15 MHz. The nonlinear coupling coefficient *g*_0_ = *ћω*_0_^2^*v*_g_^2^*n*_2_/(c*A*_eff_*L*) is related with group velocity *v*_g_, material nonlinear refractive index *n*_2_ and effective volume *A*_eff_*L*. Based on the parameter of the used HLNF, we set Kerr nonlinearity coefficient *n*_2_ = 2.4 × 10^−19^ m^2^ W^−1^ and effective mode area *A*_eff_ = 12.4 μm^2^. Inside the Fourier transform term ${{{\mathcal{F}}}}[]$, *f*_R_ indicates the Raman fraction, and *h*_*R*_ (*T*) is the Raman response. We use a modified form of Raman response proposed in ref. ^50^, and *h*_*R*_ (*T*) can be expressed as

6 $$\begin{array}{l}{h}_{R}\left( {T} \right) = \left( {{1} - {f}_{b}} \right)\left( {{\tau} _{1}^{ - 2} + {\tau} _{2}^{ - {2}}} \right){\tau} _{1}{\rm{exp}} ( - {T}/{\tau} _{2}){\sin} ({T}/{\tau} _{1})\\ \qquad\qquad \,+ \,{f}_{b}\left[ {({2}{\tau} _{b} - {t})/{\tau} _{b}^{2}} \right]{\rm{exp}} ( - {T}/{\tau} _{b})\end{array}$$

where *τ*_1_ is related to a single vibrational frequency of silica molecules and *τ*_2_ is the damping time of vibration. Here, we set *τ*_1_ = 12.2 fs and *τ*_2_ = 32 fs for silica fiber. Considering the contribution of boson peak with *τ*_b_ = 96 fs, the *f*_*b*_ = 0.21, and *f*_*R*_ = 0.245 are used in Raman term^50^. In the Fabry-Perot cavity, the nonlinear interactions between the forward- and reverse-transmission field introduce an additional nonlinear term *ig*_0_${{{\mathcal{F}}}}$[2〈|*A*|^2^〉*A*], where 〈|*A*|^2^〉 denotes the average intracavity photon flux over time domain^29,32^ and defined as

7 $$\langle {\left| {A} \right|^{2}} \rangle = \frac{1}{{{T}_{R}}}{\mathop {\int}\nolimits_{- {T}_{R}/{2}}^{{T}_{R}/{2}}} \left| {{A}\left( {{t},{T}} \right)} \right|^{2}\;{dT}$$

where *T*_R_ is the round-trip time. The pulsed pump term *F*_*μ*_ (*t*) can be described as

8 $${F}_{\mu} {(t)} = \frac{{\sqrt {{P}_{0}/{\hbar} {\omega} _{0}} }}{{{M} + {1}}}\mathop {\sum }\limits_{{\mu} = - {M}/{2}}^{{\mu} = {M}/{2}} {\delta} {({\mu} ^{\prime} - {\mu} )}$$

with the pulse peak power *P*_0_ = 4.2 W and *M* + 1 = 51 equal driving comb lines. For near-zero-dispersion soliton simulation, we use the experimental parameters of *D*_1_/2π = 10.41506 GHz, and driving pulse repetition rate *f*_rep_ = 10.415022 GHz, thus the drift coefficient *D* = − 2π × 38 kHz. As the dispersion measurement of *d*_int_ inevitably introduces errors in *D*_2_ and *D*_3_ (Supplementary Section 6), we choose the values of *D*_2_ and *D*_3_ near the measured values so that the simulation can best re-produce the experimental results. The chosen values are *D*_2_/2π = 40.6 Hz, and *D*_3_/2π = −0.7 Hz. The parameter normalization in Eq. (1) follows that of ref. ^51^: *t*' = *κt*/2, *τ* = $D_1T\left| {\frac{\kappa }{{D_2}}} \right|^{1/2}$, Ω = $\frac{{2\pi }}{\tau }$, *ζ*_0_ = 2*δω*/*κ*, *d* = $D\frac{2}{\kappa }\left| {\frac{\kappa }{{D_2}}} \right|^{1/2}$, *d*_1_ = $\frac{{2D_1}}{\kappa }\left| {\frac{\kappa }{{D_2}}} \right|^{1/2}$, *d*_3_ = $- \frac{{D_3}}{{3\kappa }}\left| {\frac{\kappa }{{D_2}}} \right|^{3/2}$, *ψ* = $A\left| {\frac{{2g_0}}{\kappa }} \right|^{1/2}$, *F* = $P_0\frac{{8\kappa _{ext}g_0}}{{\kappa ^3\hbar \omega _0}}$. Here, cavity decay rate *κ* is set as *κ* = *κ*_0_ + *κ*_*ext*_ (μ = 0).

### F-P microresonator fabrication

Our F-P microresonator is made of a single-mode HNLF with a weak anomalous GVD near 1550 nm. The HNLF is mounted in a ceramic ferrule for protection and support. Two facets of the fiber are then polished and coated with 15 pairs of Ta_2_O_5_ and SiO_2_ layers to form a Bragg mirror with a reflection rate >99.8% near 1550 nm. The thickness of the film is about 7 μm, which is uniform and would not introduce additional dispersion to the microresonator. Different from the F-P microresonator demonstrated in the works of Obrzud^29^, we use a ceramic ferrule with a 1.25 mm diameter for better heat dissipation and temperature control. The fabricated F-P microresonator is shown in the top inset of Fig. 2a.

### Experimental setup

In the setup, the light source is generated by a 1550-nm CW laser (NKT, E15) with fast wavelength modulation range up to 8 GHz. Three phase modulators (EOSPACE, PM) are used to provide periodic chirp and an intensity modulator (EOSPACE, IM) is used to modulate the linear chirp region of the field. The linearly chirped field is then compressed by a dispersion compensation module (DCM) and generates a picosecond pulse train^52^. The total modulation index *θ* = 10.6π, and the dispersion of the DCM is −16.023 ps nm^−1^. We use an optical sampling oscilloscope of 500 GHz bandwidth (Alnair-lab, EYE-2000C) to monitor its temporal profiles in real-time, and the pulse duration is fitted to be 2.1 ps.

In the experiment, the F-P microresonator is fiber coupled by a standard fiber connector. We specially design a metal mount to hold the microresonator and a TEC module is placed under the metal mount to stabilize the temperature of the F-P microresonator. The silica fiber exhibits a positive thermo-optic coefficient of d*n*/d*T* ≈ 1.09 × 10^−5^/°C at room temperature and C-band wavelength range^53^. In the case of resonator FSR = ~10 GHz, the temperature-induced FSR variation can be calculated as dFSR/d*T* ≈ 100 kHz °C^−1^, which is consistent with our measurement results. Therefore, we can tune the desynchronization frequency *δf*_rep_ by setting the operating temperature of the TEC module, which can significantly reduce the difficulty of the experiment. The TEC module’s temperature control accuracy is 0.01 °C, which corresponds to an FSR control accuracy of ~1 kHz.

## Supplementary information

Supplementary GIF for fig. S1

Supplementary Information for Near-zero-dispersion soliton and broadband modulational instability Kerr microcombs in anomalous dispersion
