# Supplementary figures and images for: Near-zero-dispersion soliton and broadband modulational instability Kerr microcombs in anomalous dispersion
Source: Light Sci Appl. 2023 Feb 1;12:33. doi: 10.1038/s41377-023-01076-8 (PMC9892599; doi:10.1038/s41377-023-01076-8)

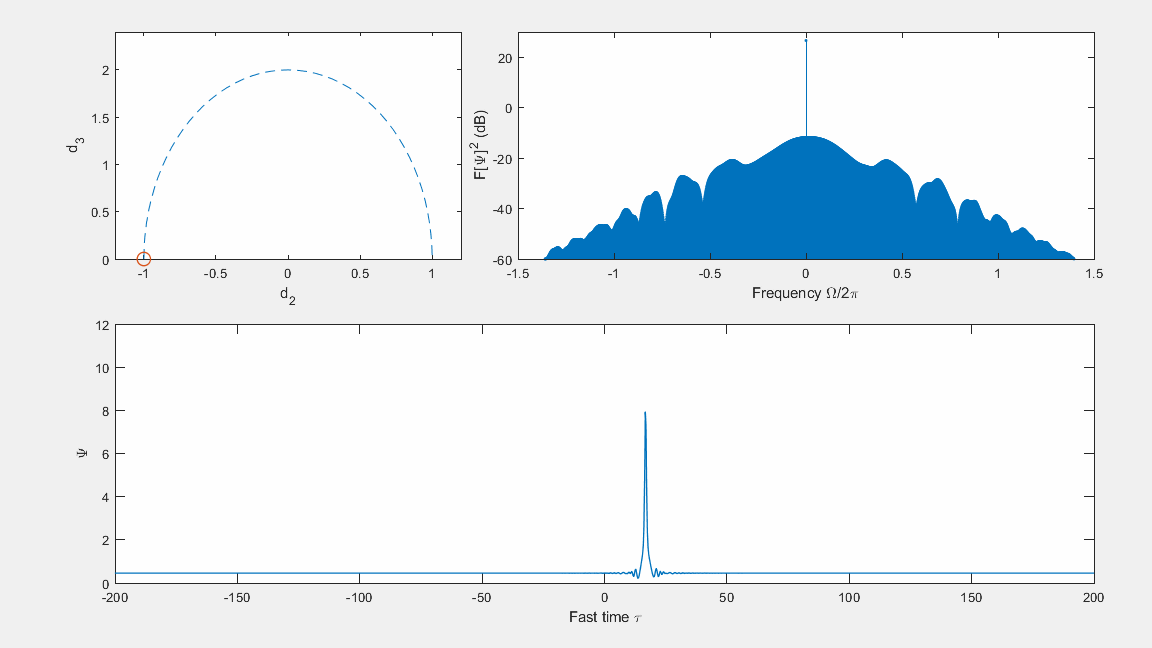

Supplement: Supplementary file 1 — Supplementary GIF for fig. S1 [file 41377_2023_1076_MOESM1_ESM.gif]
