# Supplementary material for: Near-zero-dispersion soliton and broadband modulational instability Kerr microcombs in anomalous dispersion
Source: Light Sci Appl. 2023 Feb 1;12:33. doi: 10.1038/s41377-023-01076-8 (PMC9892599; doi:10.1038/s41377-023-01076-8)
Supplement: Supplementary file 2 — Supplementary Information for Near-zero-dispersion soliton and broadband modulational instability Kerr microcombs in anomalous dispersion [file 41377_2023_1076_MOESM2_ESM.docx]

Supplementary Information for

**Near-zero-dispersion soliton and broadband modulational instability Kerr microcombs in anomalous dispersion**

Zeyu Xiao^1^†, Tieying Li^1^†, Minglu Cai^1^, Hongyi Zhang^1^, Yi Huang^1^, Chao Li^1^, Baicheng Yao^2^*, Kan Wu^1^* and Jianping Chen^1^

^1^ State Key Laboratory of Advanced Optical Communication Systems and Networks, School of Electronic Information and Electrical Engineering, Department of Electronic Engineering, Shanghai Jiao Tong University, Shanghai 200240, China

^2^ Key Laboratory of Optical Fibre Sensing and Communications (Education Ministry of China), University of Electronic Science and Technology of China, Chengdu 611731, China.

^*^Corresponding author e-mail address: [yaobaicheng@uestc.edu.cn](mailto:yaobaicheng@uestc.edu.cn), [kanwu@sjtu.edu.cn](mailto:kanwu@sjtu.edu.cn)

† These authors contributed equally to this work.

1. Near-zero-dispersion soliton dynamics with varied dispersion

The Supplementary GIF for Fig. S1 shows the corresponding near-zero-dispersion soliton (NZDS) dynamics. Fig. S1 depicts the physical relation between the conventional single DKS and the proposed AD-NZDS, as the resonator dispersion follows an elliptical path in the second-order / third-order dispersion (*d*_2_/*d*_3_) coordinate. The simulation is performed based on the normalized Lugiato-Lefever Equation for Fabry-Pérot resonator (FP-LLE), which is given by

|  | $\frac{\partial\psi}{\partial t^{'}}=\left( -id_{2}\frac{\partial^{2}}{\partial\tau^{2}}+d_{3}\frac{\partial^{3}}{\partial\tau^{3}} \right)\psi+\left( i\left\vert\psi\right\vert^{2}+2i\left\langle\left\vert\psi\right\vert^{2} \right\rangle-i\zeta_{0}-1 \right)\psi+\sqrt{F_{0}}$ | (S1) |
| --- | --- | --- |


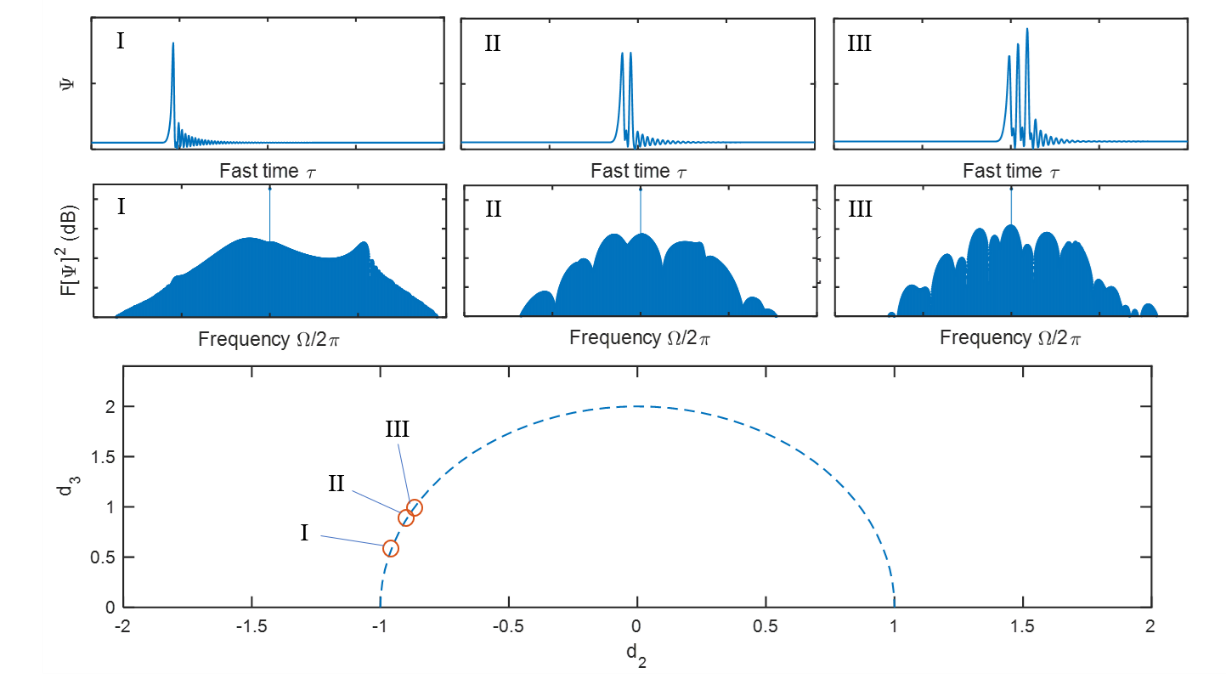


**Fig. S1. AD-NZDS evolution in a *d*_2_/*d*_3_ coordinate with a continuous-wave pump.** The orange circles in bottom figure indicate **I** dissipative Kerr soliton with dispersive-wave tails, **II** two-soliton bonding state, and **III** three-soliton bonding state, respectively. Extended version of this figure with a GIF available in Supplementary Info.

In contrast to Eq. 1 of the main text, this equation is driven by a CW light with a power of *F*_0_, and does not include the field drift term. Other symbol definitions can be found in the theoretical analysis section of the main text. In the simulation, second-order and third-order dispersion (*d*_2_ and *d*_3_) changes over the loop number *n*_L_ (*n*_L_ is an integer), where *d*_2_ (*n*_L_) = −cos(2π*n*_L_/*N*_L_) and *d*_3_ (*n*_L_) = 2sin(2π*n*_L_/*N*_L_) with *n*_L_ ∈[0, 147] and the total loop number *N*_L_ = 1000. In each loop, the intracavity field runs 256 round trips to provide enough evolution time. The model is hard-excited by an initial single DKS with CW driving power of *F*_0_ = 10, and detuning of ζ_0_ = 5.9. The intracavity field *ψ*(τ) is initially in the breathing state, as shown in the Supplementary GIF for Fig. S1. When TOD dominates the cavity field, the breathing soliton gradually stabilizes and temporally drifts to the right. With the further increase of TOD, the second sub-soliton is spontaneously excited at the oscillatory tail of the leading soliton, and forms a bound soliton structure of NZDS^(2)^. Subsequently, NZDS^(3)^ and NZDS^(4)^ are also observed, before the multi-peak structure is destroyed by intracavity modulational instability (MI). Our simulations demonstrate that the NZDS theory is equally applicable in both CW driving and pulsed driving scenarios.

1. Continuous decrease of the number of NZDS

In this section, we have discussed the mechanism of the one by one decrease of the pulse number in the near-zero-dispersion soliton. The simulation was performed in the normalized FP-LLE, and the simulation parameters are consistent with the main text. Fig. S2a presents the power transmission with the increase of pump detuning, and Fig. S2b exhibits the corresponding intracavity temporal evolution. Under the combined effects of third-order dispersion and the desynchronization of the driving pulse, the intracavity field possesses negative group-velocity shift, as shown in the Fig. S2b. That is, the center of the soliton cluster shifts along the τ >0 direction. Three NZDS states with 10, 9 and 8 bound solitons are marked as I-III in Fig. S2a and S2b and their waveforms are shown in Fig. S2c. The corresponding detuning values are chosen at the end of each soliton steps so that we can obtain the temporal profiles just before the number of the soliton is switched. The orange curves in Fig. S2c represent the profiles of intracavity pulsed pump with their peak power calculated by the steady-state solution of the FP-LLE [S1]. Since the proposed near-zero-dispersion solitons are generated at the bistable lower branch, the power of the intracavity pulsed pump decreases with the increase of pump-resonance detuning. As the soliton cluster drifts towards the positive direction in the moving frame of the driving pulse, the rightmost soliton will disappear first due to the insufficient pump power. The lowest intracavity power requirements is simulated to be 0.15, as shown in Fig. S2c. Therefore, the number of solitons will decrease one by one with the increase of detuning.


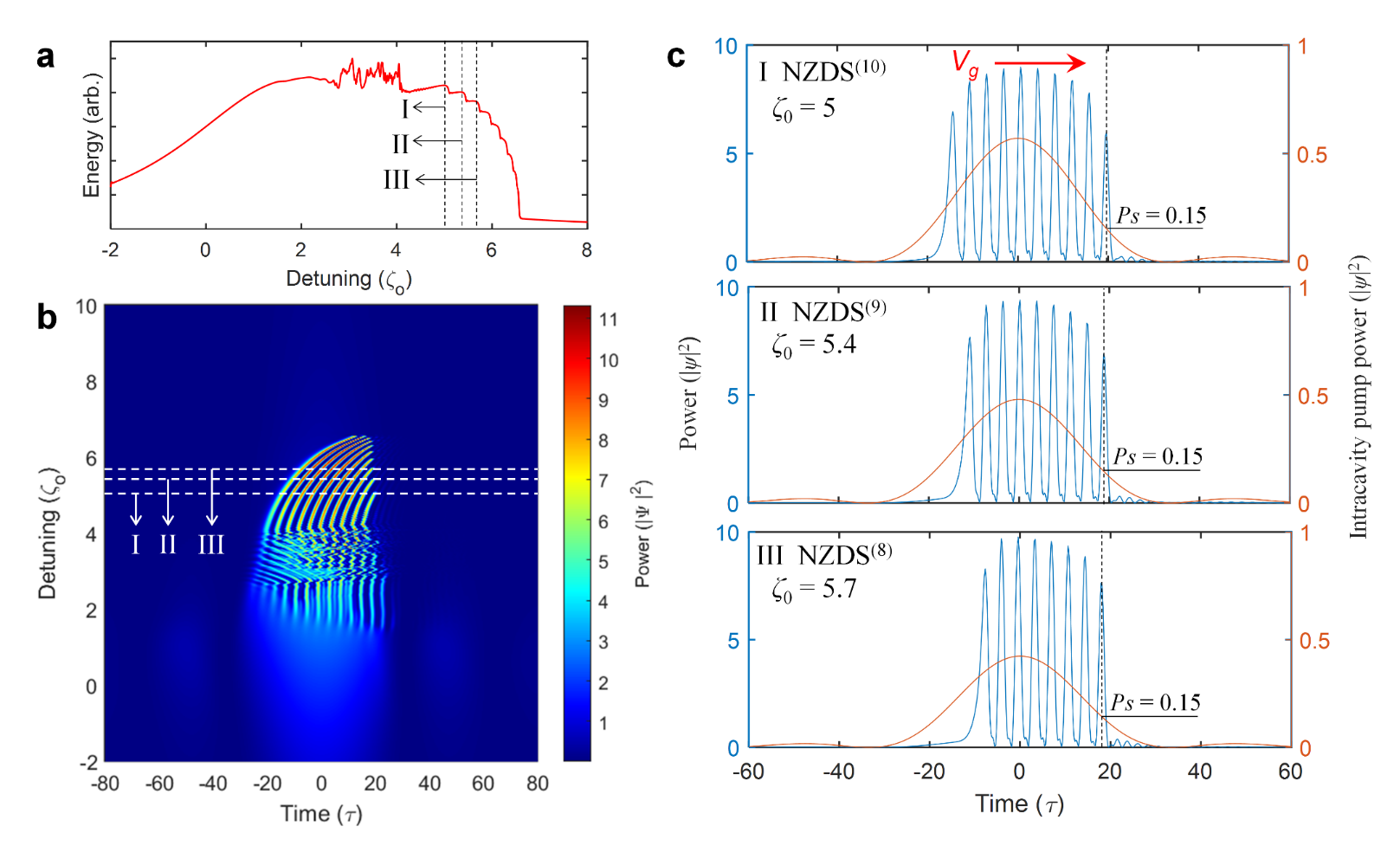


**Fig. S2. Pulse number continuously decrease in the near-zero-dispersion soliton. a** Intracavity power variation as the detuning increases from −2 to 8. **b** The corresponding temporal waveform evolution. **c** The waveform profiles of the near-zero-dispersion soliton (blue curve) and the intracavity pulsed pump (orange curve) with the detuning *ζ*_0_ = 5, 5.4 and 5.7, respectively.

1. Near-zero-dispersion soliton simulation with experimental parameters

The experimental generation of near-zero-dispersion soliton can be simulated with the Lugiato-Lefever equation for Fabry-Perot resonator (FP-LLE). We include the Raman terms and frequency-dependent coupling loss in the FP-LLE because of the ultra-wide spectrum of NZDS. In the simulation, we use simulated reflection parameters of the F-P resonator, as shown in Fig. S3a. Other simulation parameters are similar to those used in our experiment, as described in the Materials and Methods of the main text. We demonstrate the simulation result of the NZDS formation in Fig. S3c and S3d, and the corresponding spectral and temporal profiles are given in Fig. S3e and S3f. During the frequency scan of the driving pulse, four typical states can be obtained: the primary comb state (Fig. S3e I and S3f I), the broadband MI comb state (Fig. S3e II and S3f II), the unstable soliton state (Fig. S3e III and S3f III) and the soliton state (Fig. S3e IV and S3f IV), which is similar to the conventional dissipative Kerr soliton (DKS) generation process. However, due to intracavity modulational instability (MI) and relatively strong third-order dispersion, we obtain a regularly arranged multi-soliton structure in the soliton state. The generated multi-solitons collapse one by one with the increasing of the detuning as indicated by the steps feature in Fig. S3b. The gray dashed lines in Fig. S3b correspond to the detuning positions of the simulated spectra of NZDS^(12)^, NZDS^(11)^ and NZDS^(10)^, as shown in Fig. 5(d) of the main text. The calculated pump-to-comb conversion efficiency is also exhibited in Fig. S3b. The average conversion efficiency of the broadband MI state is 8.7%, while that of the NZDS state is from 3.8% - 5.7% (corresponding to NZDS^(6)^ - NZDS^(13)^ states). Obviously, the MI state has a higher conversion efficiency than the NZDS state. The spectral evolution in Fig. S3d gives some insight about the origins of NZDS. The spectrum of NZDS is characterized by prominent optical comb envelopes on two sides of the driving modes, which are evolved from the primary comb envelopes, as marked in Fig. S3c. It is well known that third-order dispersion enables the stabilizing of intracavity fields [S2], and the spectral structure of the residual primary comb can be stabilized by TOD. Eventually, such a binding structure of soliton clusters can be formed in the temporal domain.


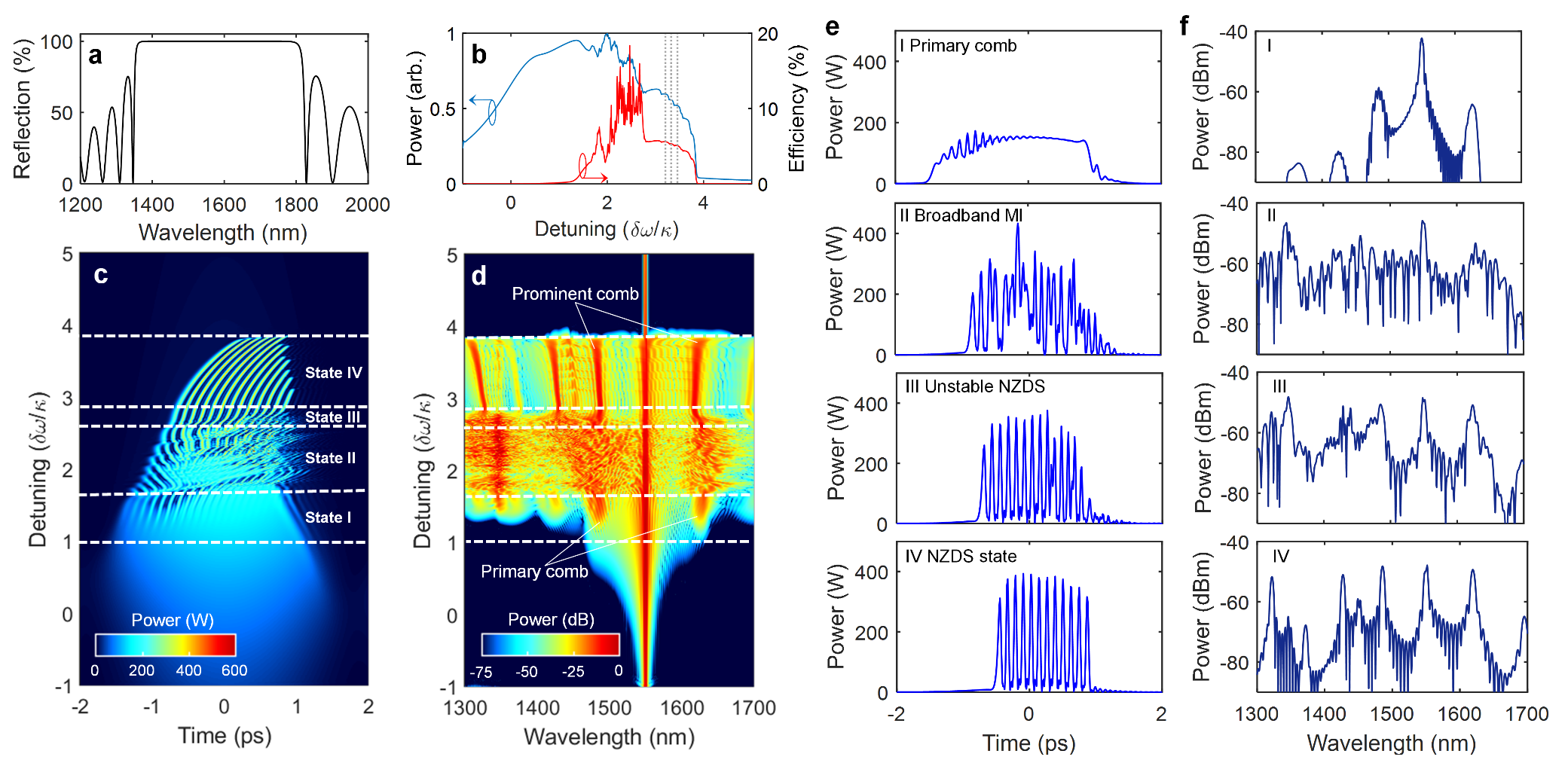


**Fig. S3. Full model of FP-LLE for simulation. a** Simulated reflectance spectrum of the mirror coatings in the F-P microresonator. **b** Power variation (blue line) and pump-to-comb conversion efficiency (red line) versus increasing detuning. **c** Intracavity temporal waveforms and **d** simulated output spectra using the experimental parameters. States I-IV correspond to the primary comb, broadband MI, unstable NZDS, and NZDS state, respectively. The corresponding temporal and spectral profiles are shown in **e** and **f**, and their detuning are I *δω*/*κ* = 1.4, II *δω*/*κ* =2.5, III *δω*/*κ* = 2.7, and IV *δω*/*κ* = 3.2, respectively.

The microcomb evolution is simulated in the full FP-LLE model with small anomalous SOD and relatively large TOD, as well as including the Raman effect and wavelength-dependent coupling loss of the dielectric mirror coating. To explore their impacts on the soliton structure, we remove the TOD term, Raman response term, and wavelength-dependent coupling loss term from the FP-LLE model one at a time. Fig. S4 depicts four sets of simulation results, and the simulation parameters are similar except for the comparison terms mentioned above. Fig. S4a presents the simulated output spectrum and intracavity temporal waveform only considering the intracavity SOD, and the mode-resonance detuning *δω*/*κ* = 3.2. We obtain conventional multi-soliton state, that exhibiting 18 separate pulses with arbitrary time intervals at the top of the driving pulse. This suggests that the NZDS structures and the resonator TOD are closely related. The resonator TOD is then included in the model, with the corresponding results displayed in Fig. S4b. However, the NZDS structures are not observed in the temporal profiles (see bottom panel of Fig. S4b). Instead, a hybrid-binding structure of single soliton and multi-peak soliton is found. The explanation for this phenomenon is that the resonator TOD is too large, and the calculated normalized *d*_3_ /*d*_2_ = 5. Due to the stabilization mechanism provided by the strong TOD [S2], the intracavity field is not fully modulated by the MI, which results in partially separated sub-soliton structures being bound in soliton structures as well. In contrast, when TOD is comparable to SOD (e.g., the case of *d*_3_ /*d*_2_ = 1 in Fig. 2 of the main text), MI permits the sub-soliton structure to be completely separated, generating the expected NZDS structures. The observed hybrid structure is also reported for the first time, and a discussion of this structure is beyond the scope of this paper. We further add the Raman response term in the FP-LLE model, and the simulation results are given in Fig. S4c. Despite the resonator having a significant TOD, the pure NZDS structure is observed with the assistance of Raman effect. We find that the primary comb envelope on the long-wavelength side is just near the fiber Raman gain spectrum (Raman frequency shift of 11.6 THz), which effectively enhances the intracavity MI and enables the full modulation of sub-soliton. It should be noted that while the NZDS generation does not rely on the Raman effect, the Raman effect can help the NZDS generation in resonators with large TOD. Finally, the wavelength-dependent coupling loss of the mirror coating is included in the model, and the simulation results are shown in Fig. S4d. The output spectrum of the resonator will be shaped by the transmission spectrum of the mirror coating, and the modeling predictions are in good agreement with the experimental results, as shown in Fig. 4d of the main text.


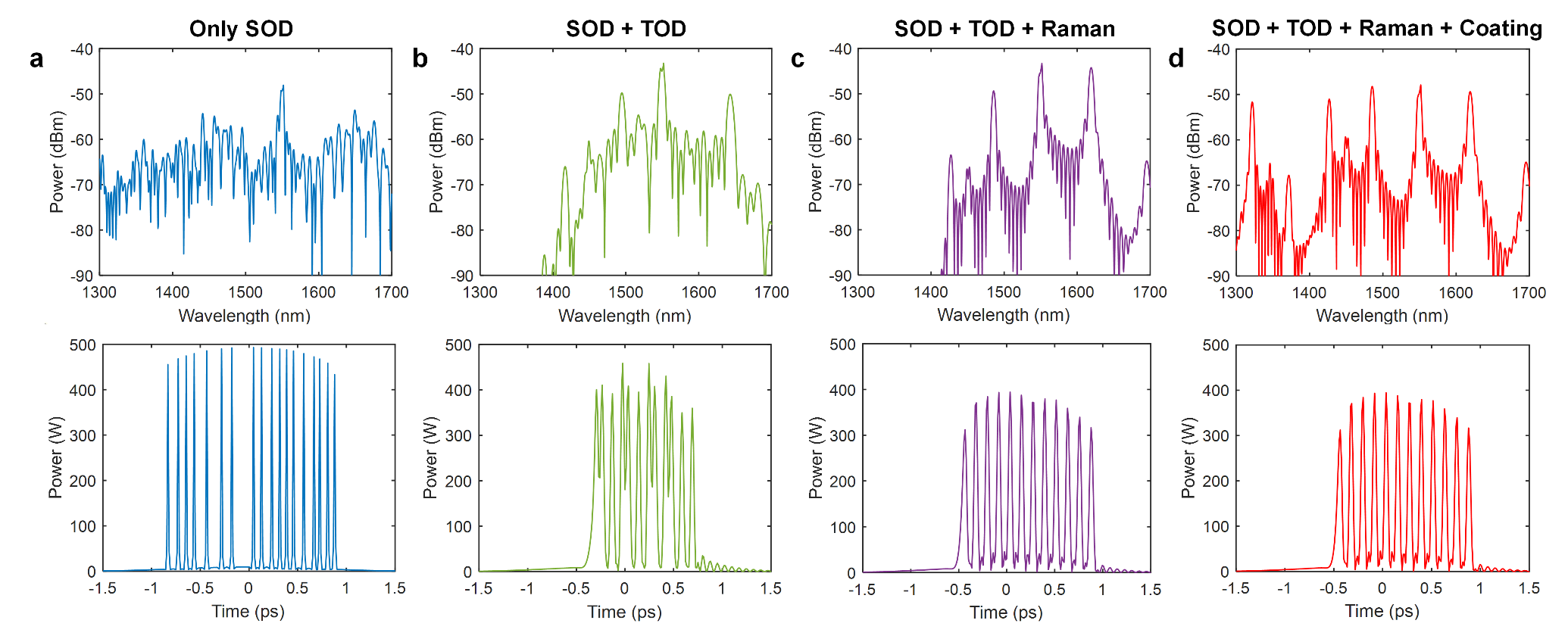


**Fig. S4. Simulation investigation of the roles of SOD, TOD, Raman and wavelength-dependent transmission.** The simulation results of spectral and temporal profiles in the cases including **a** only SOD; **b** SOD and TOD; **c** SOD, TOD and Raman; **d** SOD, TOD, Raman and wavelength-dependent transmission of the mirror coating.

To examine the coherence of the generated microcombs, we calculate the coherence function *g*_12_, which is defined by

$$\left|g_{12}^{(1)}(\lambda,t_{1}-t_{2})\right|=\left|\frac{\langle\widetilde{A}_{1}^{*}(\lambda,t_{1})\widetilde{A}_{2}(\lambda,t_{2})\rangle}{\sqrt{\langle|\widetilde{A}_{1}(\lambda,t_{1})|^{2}\rangle\langle|\widetilde{A}_{2}(\lambda,t_{2})|^{2}\rangle}}\right|$$

where the angle brackets indicate the ensemble average over pairs of spectra $\widetilde{A}_{1}^{*}(\lambda,t_{1})$ and $\widetilde{A}_{2}(\lambda,t_{2})$ from a large number of simulations [S3, S4], and *t* denotes the independent measuring time. The intracavity noise is modeled by the addition of one photon with a random phase on each spectral mode. In our simulation, the *g*_12_ is calculated based on 50 individual simulated spectra of MI microcombs (*δω*/*κ* = 2.6) and NZDS microcombs (*δω*/*κ* = 3.2). The top panels of Fig. S5a and S5b show the ensemble spectra of the obtained MI microcombs and NZDS microcombs, while the bottom panels present the calculated coherence results. It is clear that there are significant spectral fluctuations in the MI microcombs state, and only the small region around the pump remains coherent. In contrast, the NZDS microcombs exhibit negligible spectral variation, and have unity coherence over a wide spectral range, except the region away from the pump.


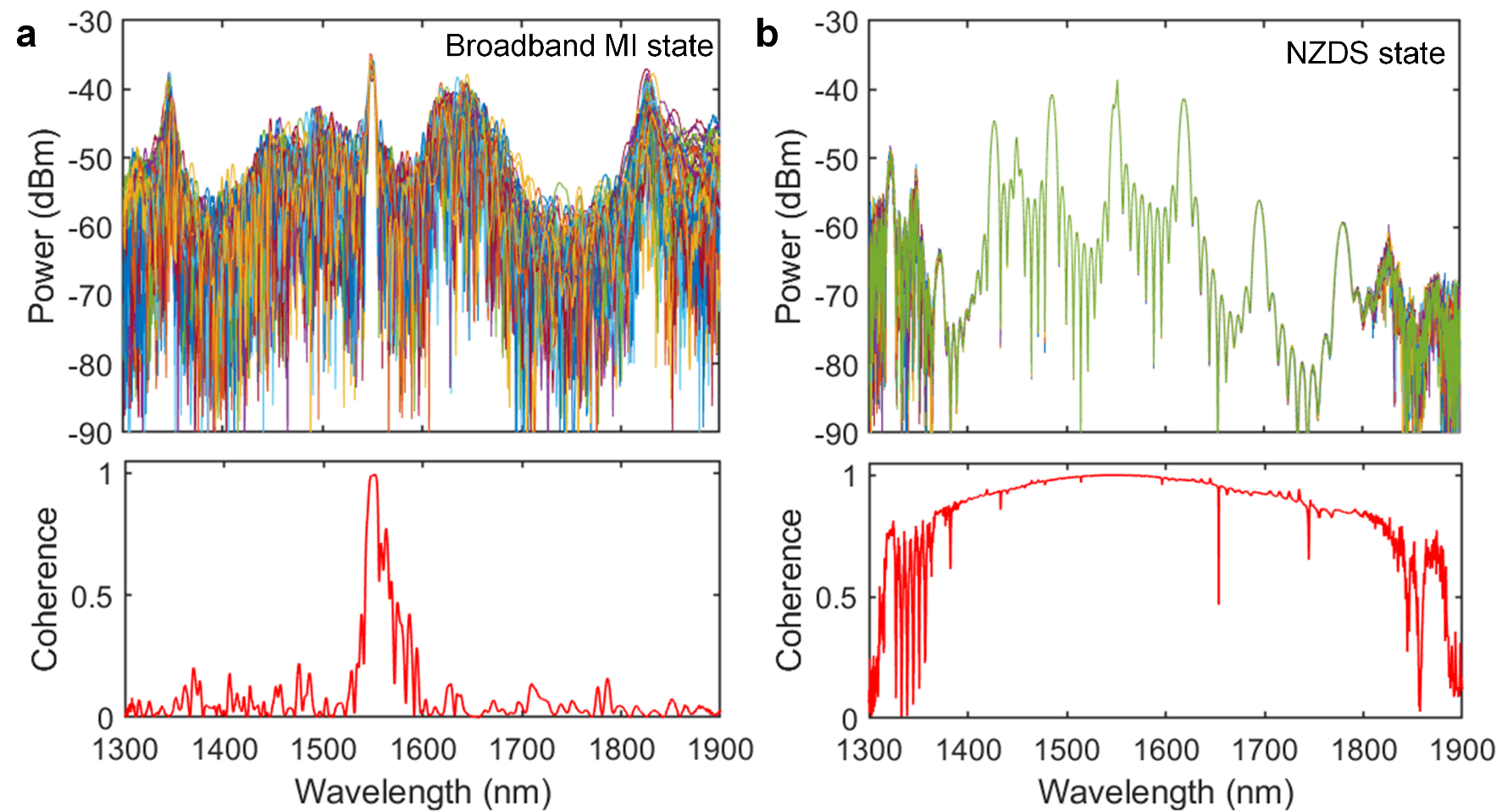


**Fig. S5. Coherence investigation of broadband MI microcomb and NZDS states.** Output spectra and calculated coherence of **a** broadband MI state and **b** near-zero-dispersion soliton state. Top panels: the ensemble spectra from 50 individual simulations. Bottom panels: the calculated coherence.

1. Group-velocity compensation

In this section, we discuss the maximum number of pulses in the NZDS and the NZDS position versus the driving pulse when varying the desynchronization frequency *δf*_rep_. Other simulation parameters are consistent with NZDS in the Materials and Methods section. Fig. S6a presents the cavity power transmission trace for the *δf*_rep_ ranges from 0 kHz to −100 kHz. The soliton steps feature is observed only when *δf*_rep_ < 0, ranging from *δf*_rep_ = –16 kHz to *δf*_rep_ = –45 kHz. The detailed temporal field evolutions are shown in Fig. S6b i-iv, which corresponds to the *δf*_rep_ of 0 kHz, −20 kHz, −40 kHz, and −60 kHz respectively. It can be seen that the length of the soliton step gradually increases as the value of *δf*_rep_ decreases, the longest soliton step can be obtained at *δf*_rep_ = –45 kHz. When *δf*_rep_ decreases further, the stable soliton steps will disappear. This phenomenon can be understood by the following process. The intracavity TOD breaks the symmetry of the temporal and spectral profiles, which leads to a constant group-velocity shift to the soliton [S5, S6]. For the parameter under study, the TOD effect contributes a negative group-velocity to the soliton, and need a negative desynchronization of driving pulse to compensate this temporal drift. A larger desynchronization can push the soliton trapping position closer to the center of the driving pulse, thus making it more resistant to TOD-induced temporal drift. This competition between the TOD and desynchronization can be used to explain the asymmetric unfolding of the soliton step observed in the experiment and the simulation.


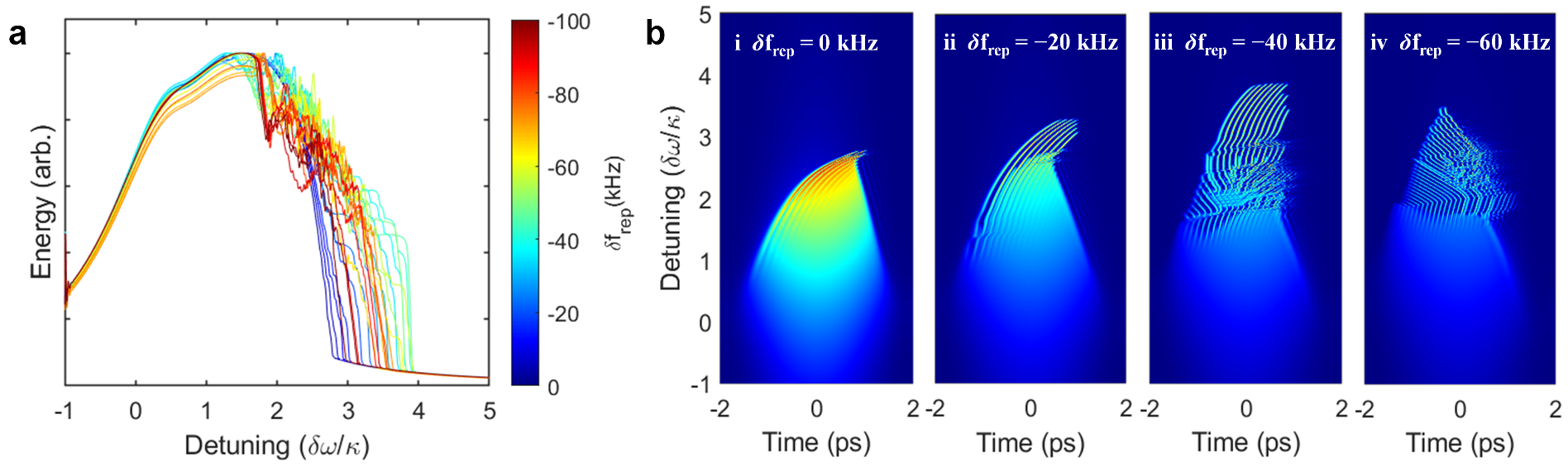


**Fig. S6. Effect of frequency desynchronization. a** Intracavity power evolution of NZDS for desynchronization *δf*_rep_ from 0 kHz to −100 kHz. **b** Detailed temporal field evolution for *δf*_rep_ = 0 kHz, −20 kHz, −40 kHz, and −60 kHz, respectively.


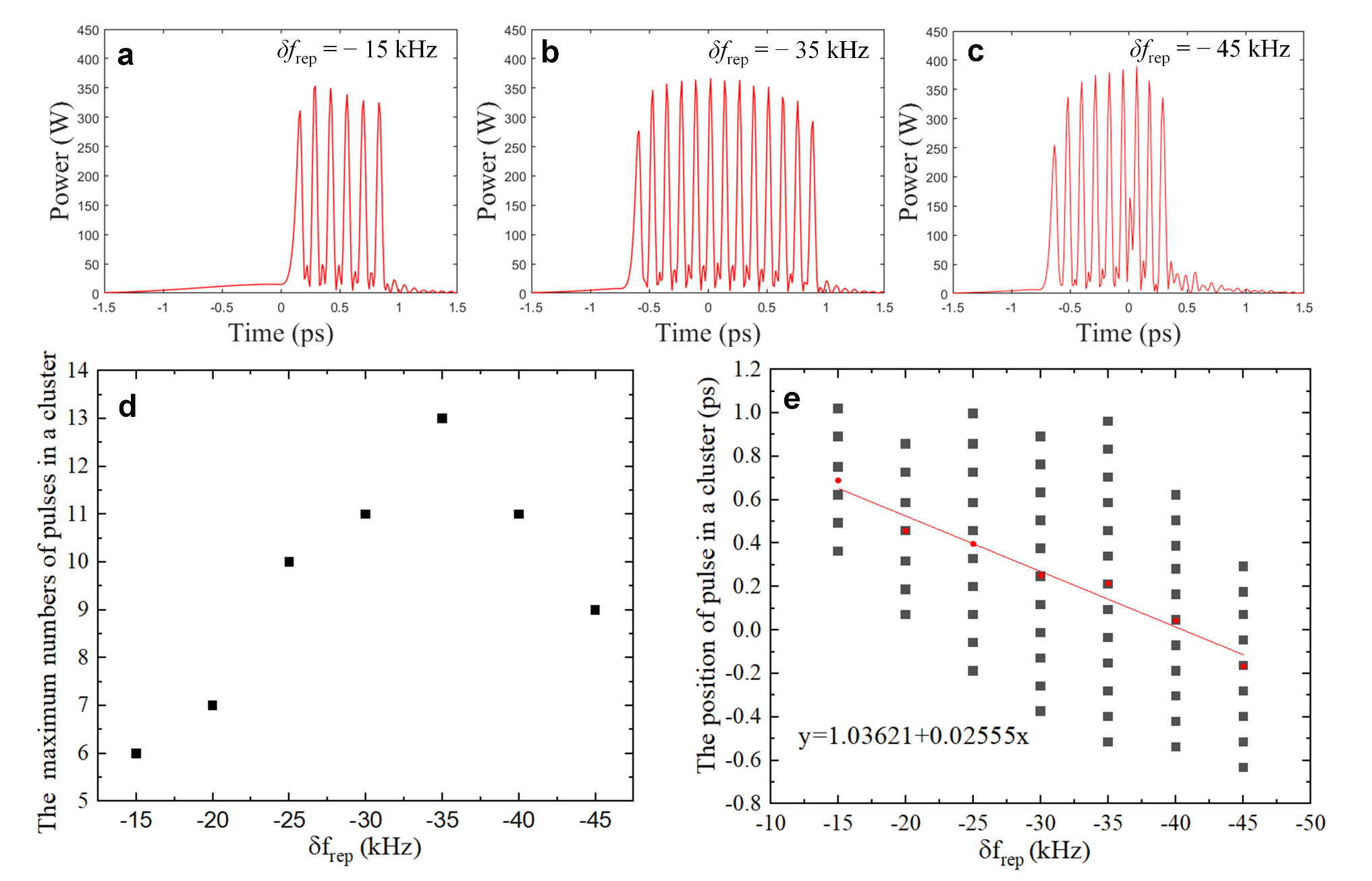


**Fig. S7**. M**aximum number and position of the pulses in a soliton cluster**. The simulation results of temporal profiles for the *δf*_rep_ of **a** −15 kHz, **b** −35 kHz, **c** −45 kHz. The numbers and the positions of pulses in a cluster versus *δf*_rep_ are shown in **d** and **e** respectively. The red dots in **e** represent the central positions of the soliton clusters.

Regarding the pulse number in a cluster and cluster position, as shown in Fig. S6, the maximum number of pulses in a cluster and the cluster position will both be influenced by the frequency desynchronization *δf*_rep_. In order to illustrate the relationship between them, we performed a further simulation study, as shown in Fig. S7. The simulation parameters are consistent with the main text but with a fixed detuning value of *δω*/κ = 3. Fig. S7a-c show the temporal waveform profiles of the soliton cluster at the *δf*_rep_ values of –15 kHz, –35 kHz, and –45 kHz. The maximum numbers of pulses in a soliton cluster versus the varied *δf*_rep_ are summarized in Fig. S7d. We can find that there exists an optimal *δf*_rep_ value of −35kHz to obtain the maximum number of 13-binding pulses. In addition, we also quantitatively analyzed the relation between the soliton cluster position and the frequency desynchronization. Fig. S7e exhibits the pulse position in a soliton cluster (black dots) and their central positions (red dots). It can be seen that the central positions of the soliton clusters almost vary linearly with the varied *δf*_rep_. After linear fitting, its drift slope is 0.02555 ps/kHz. The reason for the variation in the position of the soliton cluster can be understood as follows: The total drift velocity *v* of the soliton cluster is affected by three parameters, i.e., *v* = *v_t_* + *v_d_* +*v_p_*, where *v_t_* is a constant drift velocity introduced by third-order dispersion *D*_3_ (*v_t_*∝*D*_3_) [S7], *v_d_* is related to the frequency desynchronization *δf*_rep_ (*v_d_*∝*δf*_rep_) [S8], and *v_p_* is governed by the gradient of the driving field [S8]. When the soliton cluster is in a steady state, the drift velocity of *v* = *v_t_* + *v_d_* +*v_p_* needs to be equal to zero. In our simulation, we have a negative *D*_3_, so the soliton cluster possesses a positive group delay time (*v_t_* < 0) [S9]. In order to compensate for the velocity drift of *v_t_*, we have introduced a negative desynchronization *δf*_rep_, which corresponds to a negative group delay time (*v_d_* > 0). Besides, since the soliton cluster is located at the right side of the driving pulse, the inhomogeneity of the driving pulse gives the soliton cluster an additional negative group delay time (*v_p_* > 0) [S8]. Therefore, when we increase the value of desynchronization *δf*_rep_ (i.e., increase *v_d_*), the soliton cluster will automatically adjust its pulse number and drift to the center of the driving pulse to reduce the velocity of *v_p_*, which makes velocity *v* equal to 0 again. It should be noticed that when *δf*_rep_ is too large, it may cause instabilities to the soliton cluster [S8], as shown in Fig. S7c, and the stable soliton cluster cannot be obtained anymore if *δf*_rep_ exceeds –45 kHz.

1. High-order NZDS evolves to the single soliton

The temporal profiles of NZDS exhibit a multi-soliton binding structure in our above experimental and simulation results. It raises a natural question of whether it is possible that NZDS can evolve into a single soliton structure. We perform the simulation in full FP-LLE model (details see Materials and Methods in the main text) with varied second-order dispersion (SOD), while other simulation parameters are consistent with those in the Materials and Methods. Fig. S8a illustrates the simulated soliton numbers of NZDS states as a function of SOD and pump-resonance detuning. We find that multi-soliton NZDS structure will continuously collapse into a single soliton structure in the case of a relatively large SOD, which illustrates that the conventional single soliton structure (i.e., DKS) can be regarded as part of the NZDS, namely NZDS^(1)^. Fig. S8b and S8c show the simulated spectral and temporal profiles of the single soliton at *D*_2_ = 380 Hz, *δω*/*κ* = 3.34 (marked in orange circle in Fig. S8a, which directly evolves from the higher-order NZDS. Benefited from the weak SOD and strong TOD, the simulated single soliton has a wide spectrum of 328 nm in −40 dB, and an ultrashort pulse duration of 45.7 fs. Due to the spectral recoil caused by the dispersive wave (DW) [S5, S6] and Raman-induced self-frequency shift [S10, S11], the peak of soliton spectrum shifts towards the long-wavelength direction. Also, the dispersive wave around 1380 nm corresponds to a long oscillatory tail after the single soliton in the time domain.


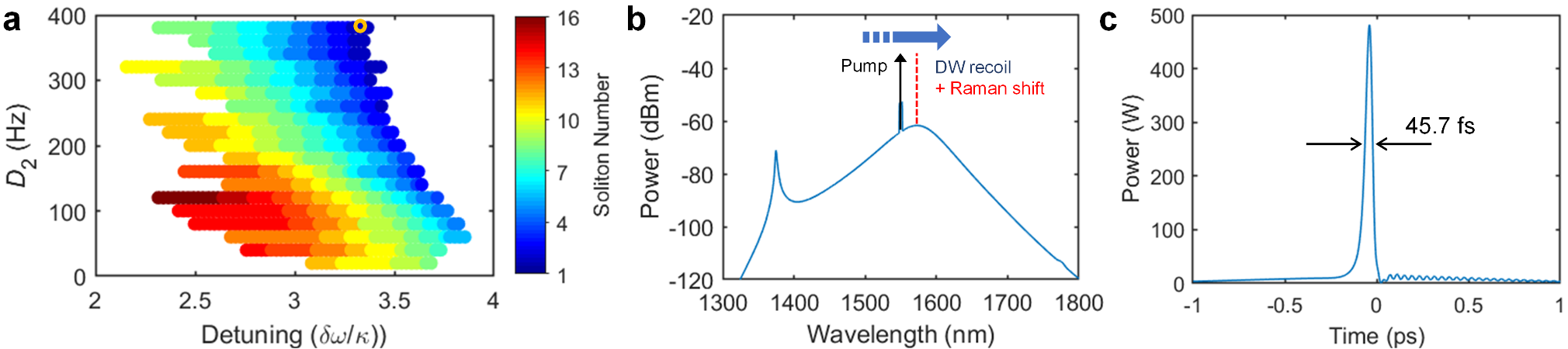


**Fig. S8. Soliton number in NZDS states. a** Soliton number with respect to detuning and second-order dispersion. **b** Simulated NZDS^(1)^ spectrum and **c** temporal waveform. Its location is marked by an orange circle in **a**.

1. FSR and dispersion measurement

Free-spectral range (FSR) and dispersion parameters are essential for both simulation and experiment of microcombs. In this section, we discuss the measurement techniques adopted to characterize the FSR and dispersion parameters of our fiber F-P microresonator.The high-accuracy measurement of FSR is critical for synchronously pulsed pump scheme. In the conventional FSR measurement with a frequency sweeping laser, the measurement accuracy is limited by the wavelength resolution and accuracy of the laser. Here, we propose an FSR measurement scheme based on a filter-driven dual-cavity structure, with a measurement accuracy of ~1 kHz. After obtaining the FSR value, the resonator dispersion is determined by using a fiber Mach-Zehnder interferometer (MZI) method [S12, S13].

The proposed FSR measurement setup is shown in Fig. S9a. The fiber-based F-P microresonator is embedded in a fiber loop cavity containing a polarization controller (PC), an Erbium-doped fiber amplifier (EDFA), a 20-km-length single mode fiber (SMF), and a band-pass filter with a bandwidth large enough to pass all oscillating modes. The purpose of band-pass filter is to control the central wavelength *λ*_0_ of the output spectrum. The F-P microresonator has a linewidth of ~10 MHz, while the mode spacing of the external fiber loop is around 10 kHz. Therefore, the proposed dual-cavity device will operate in hybrid mode, that each mode of the F-P microresonator contains thousands of modes of the external fiber loop. This process can be seen as the mode profile of the F-P microresonator being discretely sampled by the mode of the fiber loop. Fig. S9b presents the measured beatnote signal obtained by 50-GHz bandwidth photodetector, along with a Gaussian fitting curve. The resonator FSR near the central wavelength of 1550 nm is measured to be 10.41506 GHz, $\pm$5.8×10^−4^ %. It should be noted that the testing accuracy of the device is not determined by the FSR of the external fiber cavity, but by the frequency spacing of the two orthogonal modes due to fiber birefringence, which is measured to be ~1 kHz.


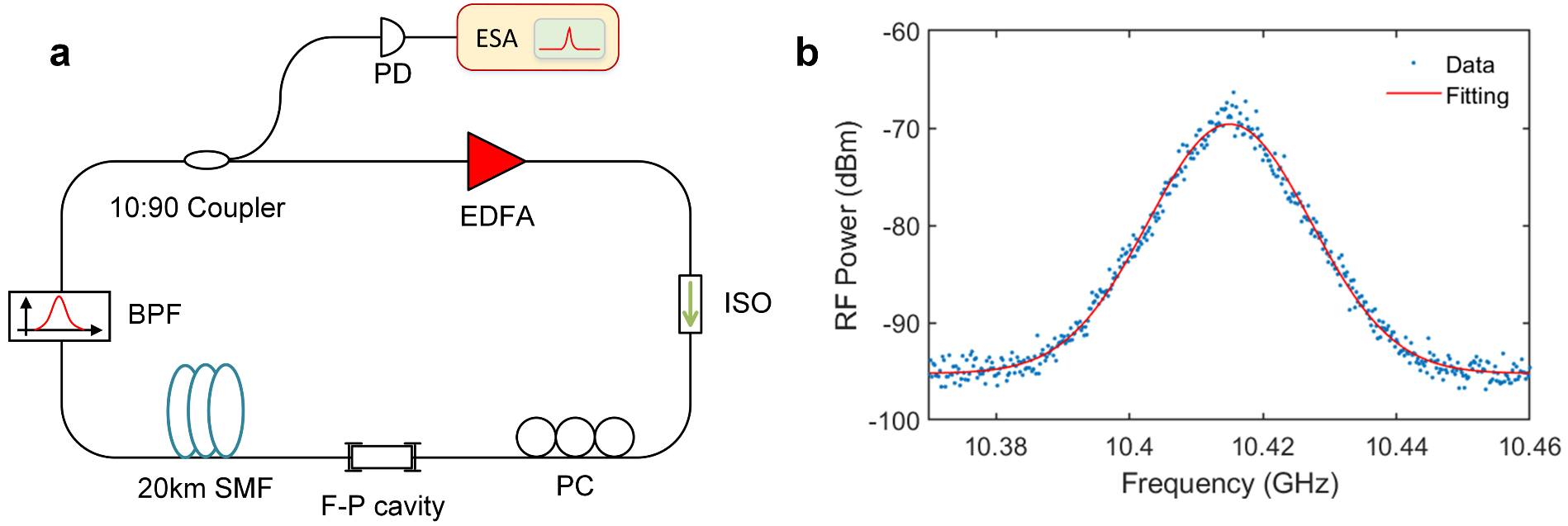


**Fig. S9. FSR measurement. a** FSR measurement setup for the F-P microresonator. PC: polarization controller, ISO: isolator, EDFA: Erbium-doped fiber amplifier, ESA: electronic spectrum analyzer, BPF: band-pass filter, PD: photodetector. **b** Beatnote signal obtain in ESA with measurement data (blue dots) and Gaussian fit (red solid).

The dispersion of F-P microresonator is measured by a fiber MZI method, as shown in Fig. S10a. The calibrated fiber MZI provides a high-accuracy and simple method to characterize the resonator dispersion without the requirement of linearity of the sweeping laser. In the experiment, the output of the tunable external-cavity diode laser (ECDL) is divided into two paths by a 3 dB coupler, and scans the F-P microresonator and the homemade fiber MZI etalon, respectively. We use 20 m-long single-mode fiber (SMF) in the upper branch of the MZI etalon to provide time delay. The tunable ECDL operates in a mode-hopping-free state from 1520 nm to 1630 nm. Fig. S10b shows the obtained time-dependent resonance signal (blue curve) and interferometer signal (green curve) in ~2 nm range, and a zoom-in view of one resonance is shown in Fig. S10c. The sinusoidal interferometer signal from MZI etalon provides calibration information that can perform time-to-frequency mapping to the resonance signal. The frequency period Δ*f*_MZI_ of the interferometer signal can be estimated by the Δ*f*_MZI_ = c/nΔ*L*_fiber_ = 10.345 MHz, where Δ*L*_fiber_ is the length difference between the two MZI branches. The frequency period of MZI can also be calibrated by the obtained FSR and the counting MZI periods near the central driving wavelength. The frequency period Δ*f*_MZI_ after calibration is 10.241 MHz. Before measuring the resonator dispersion, the dispersion of fiber MZI etalon is required to be precisely measured and calibrated since fiber itself inherently possesses dispersion. The measured dispersion of SMF used in MZI etalon is exhibited in Fig. S10d, and the dispersion calibration method can refer to [S12]. The measured integrated dispersion curve *D*_int_(*μ*) = *ω_μ_* – *ω*_0_ – *μD*_1_ is exhibited in Fig. S10e, and the result without calibration is shown in the inset. The *D*_int_ describes the deviation of the mode spacing from an equidistant frequency grid *D*_1_, where *μ* is the relative mode number of the resonance with respect to the center mode (pump mode *μ* = 0 corresponds to 1550 nm), and *D*_1_/2π is the FSR of the resonator at *ω*_0_ [S14]. The measured data are then fitted by a third-order polynomial which has a form of *D*_int_ ≈ *μ*^2^*D*_1_/2 + *μ*^3^*D*_3_/6, and obtain that *D*_2_/2π = 167 ±150 Hz, *D*_3_/2π = −1.9 ±1.3 Hz, where the measurement error is introduced in dispersion calibration of the fiber MZI. During the measurement, the MZI etalon should operate in a stable environment to avoid the perturbation in the MZI frequency period.


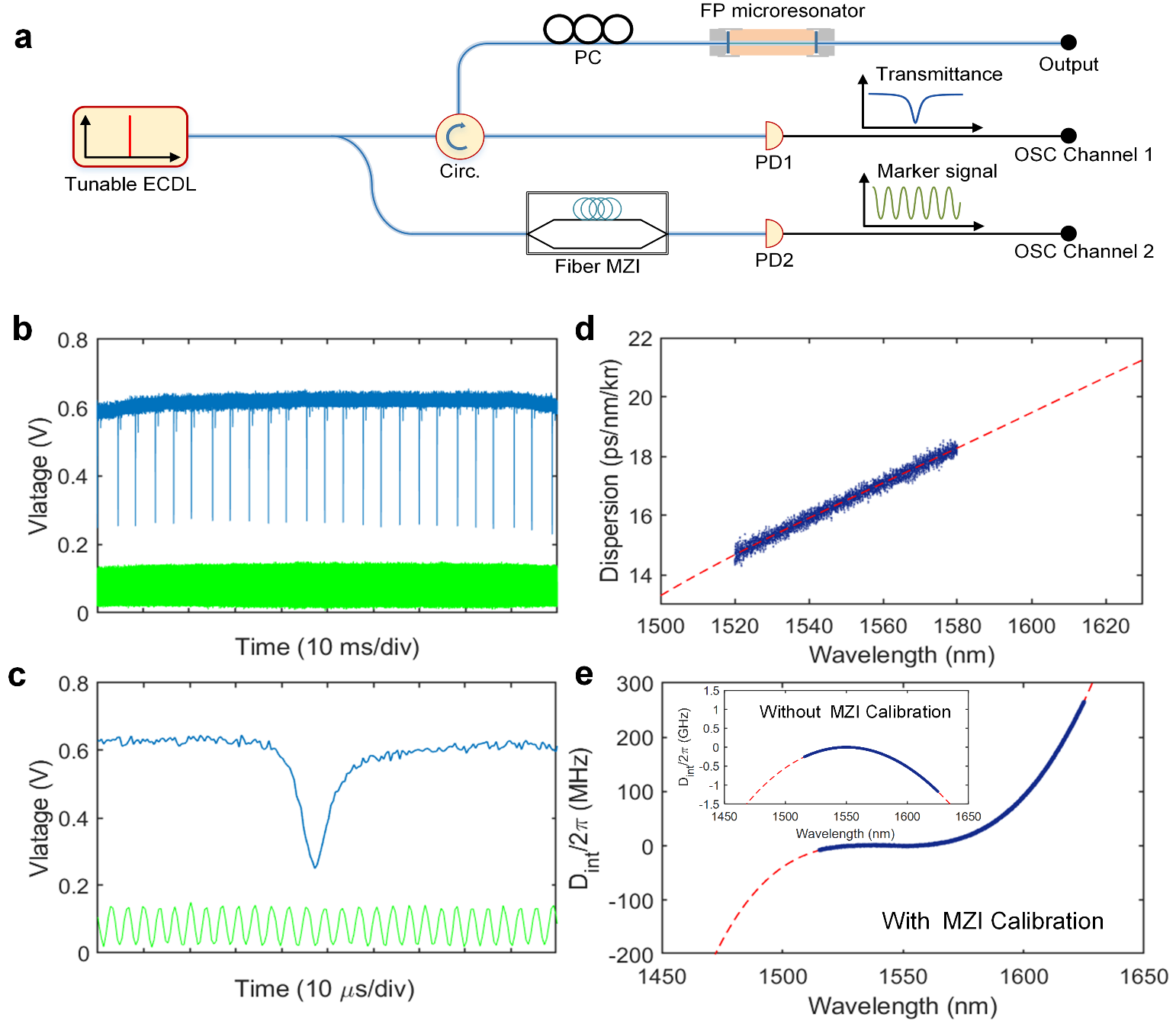


**Fig. S10. Dispersion measurement. a** Schematic of fiber MZI based dispersion measurement setup. **b** Measured time-dependent transmission spectrum of the microresonator (blue) and interferometer signal of fiber MZI (green). **c** Zoom-in view of one resonance in **b**. **d** Measured dispersion of SMF with a total length of 525m (blue dots) and fitting curve with extended wavelength (red dashed line). **e** Measured integrated dispersion *D*_int_ (blue dots) and fitting curve (red dashed line). Inset: *D*_int_ curve without MZI calibration.

1. Dispersive wave position calculation and generation of lower-order NZDS

To characterize the different stages of Kerr comb generation, we track the effective pump-resonance detuning by performing the dynamical probing of the soliton response [S15]. Due to the relatively short soliton steps, it is difficult to achieve long self-thermal locking for soliton response measurement [S16, S17]. Here, the auxiliary laser heating scheme is applied to alleviate the intracavity thermal effect, and achieve long-term soliton stability [S13]. The measurement setup is shown in Fig. S11a. We configure the auxiliary laser wavelength near 1531 nm and the pump laser wavelength near 1550 nm, and then combine the two sources by wavelength-division multiplexer (WDM) and inject them into the F-P microresonator. A fiber Bragg grating (FBG) with a bandwidth of ~ 0.2 nm and a central reflection wavelength of 1531.14 nm is used to separate the auxiliary laser signal from the pump laser signal. To ensure the effectiveness of the scheme, the wavelength tuning range of the auxiliary laser needs to be set within the highly reflective region of the FBG. In this way, PD1 is used to detect the reflected signal from the auxiliary laser, while PD2 is used to detect the reflected signal from the pump laser. In the experiment, the auxiliary laser wavelength is set in advance at the red detuned position of a resonance, while the pump laser scanned across another resonance normally from blue to the red detuned region. The scanning results are shown in Fig. S11b. It can be seen that the injected power of the auxiliary laser can effectively compensate for the intracavity power fluctuations caused by the wavelength scanning of the pump laser. When the intracavity field is stabilized at the noise state and soliton state, the corresponding transfer function curves are plotted in Fig. S11c. At the NZDS state, a clear separation of the *C*-resonance and *S*-resonance occurs, indicating the generation of high power mode-locked pulses, and the obtained effective detuning *δ*_eff_ /(2π) = 170 MHz. The spectral profile in the soliton state is shown in Fig. S11d, and features a dispersive wave at 1442 nm. The spectral location of this dispersive wave needs to satisfy the condition that the phase mismatch Δ*Φ* = *D*_int_ (*μ*) + (FSR /*f*_rep_ – 1) *D*_1_*μ* + *δ*_eff_ = 0 [S18, S19], where integrated dispersion *D*_int_ (*μ*) ≈ *μ*^2^*D*_2_/2 + *μ*^3^*D*_3_/6. The microresonator dispersion is determined by a fiber MZI method discussed in Section 6 [S12, S13], and has *D*_2_/2π = 167 ±150 Hz, *D*_3_/2π = −1.9 ±1.3 Hz. Due to the limited precision of the dispersion measurement, we modified the dispersion data to *D*_2_/2π = 40.6 Hz and *D*_3_/2π = −0.7 Hz so that the simulation can better re-produce the experimental results as explained in the Materials and Methods in the main text. Fig. S11e shows the phase mismatch Δ*Φ* for the desynchronization value of *δf*_rep_ = –38 kHz. The position of the phase-matched DW is calculated to be 1449 nm. Although the predicted DW positions deviate from the experimental results around 7 nm, it can be explained by the fact that higher-order dispersion is not considered.


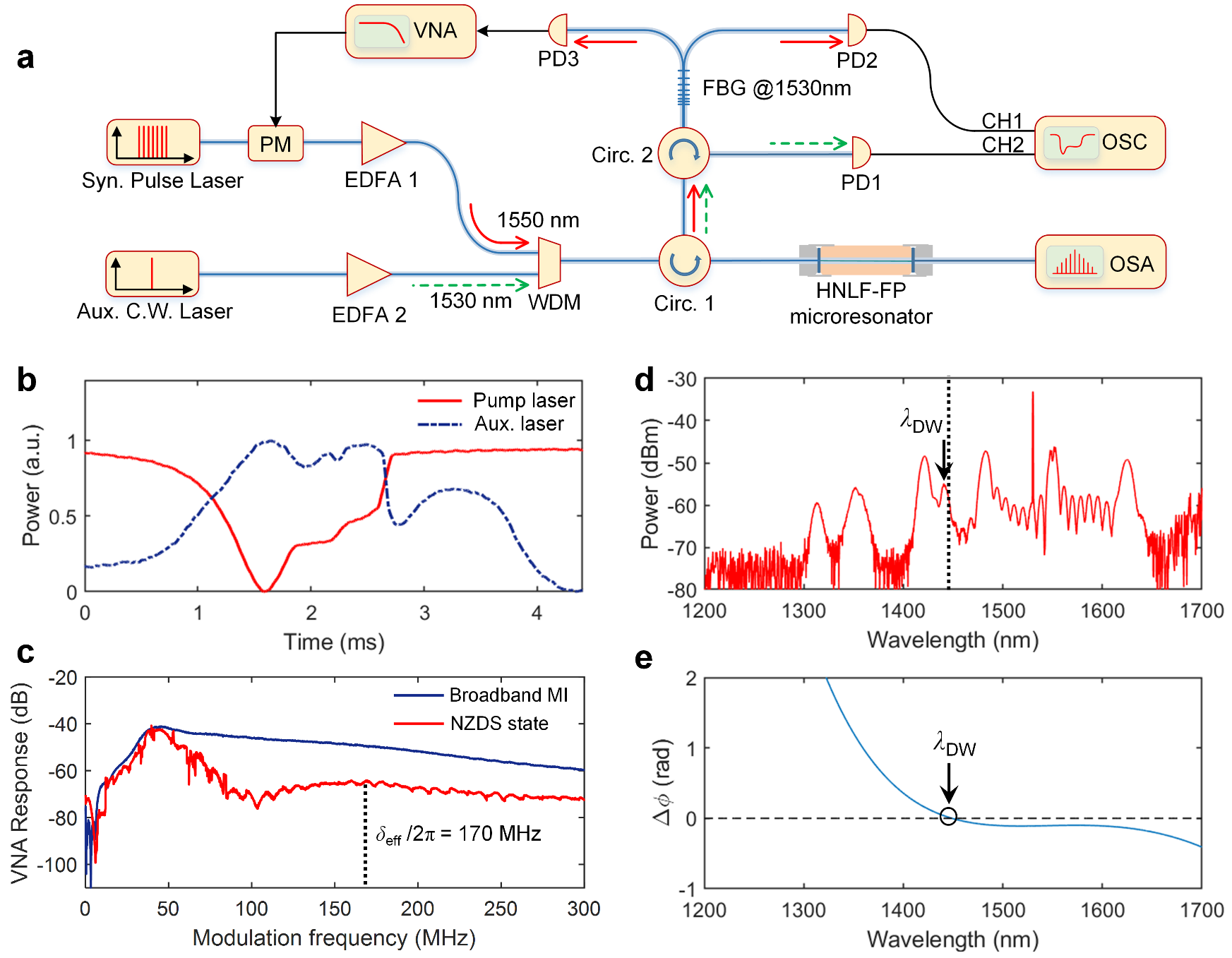


**Fig. S11. Detune measurement and dispersive wave calculation.** **a** Auxiliary laser-assisted measurement setup for dynamical probing of the soliton response. WDM: wavelength-division multiplexer; FBG: fiber Bragg grating; VNA: vector network analyzer; OSC: oscilloscope; OSA: optical spectrum analyzer. **b** Reflective signal of the pump laser (red solid curve) and auxiliary laser (blue dashed line). **c** Experimental resonance response at broadband MI state (blue) and NZDS state (red). **d** Experimental spectrum of NZDS^(8)^ state. The measured DW position is marked with a black arrow, while the theoretically predicted DW position is marked with a black dashed line. **e** Phase mismatch curve for pump desynchronizations of –38 kHz.

Moreover, we successfully obtain the lower-order NZDS^(n)^ states in our experiment with the help of the auxiliary lase, and their spectral profiles are shown in Fig. S12. The orders of NZDS^(n)^ can be identified by the spectral periodicity between the pump and prominent comb envelopes. In Fig. S12a, the soliton state is NZDS^(2)^ since there is no minor coherent envelopes between the pump and prominent comb envelopes, while in Fig. S12b, the soliton state is NZDS^(3)^ since there is one minor coherent envelopes between the two prominent comb envelopes. We also identify the obvious DW envelopes in the spectra of NZDS^(2)^ and NZDS^(3)^, which are marked by the red arrow in Fig. S12a and S12b, respectively.


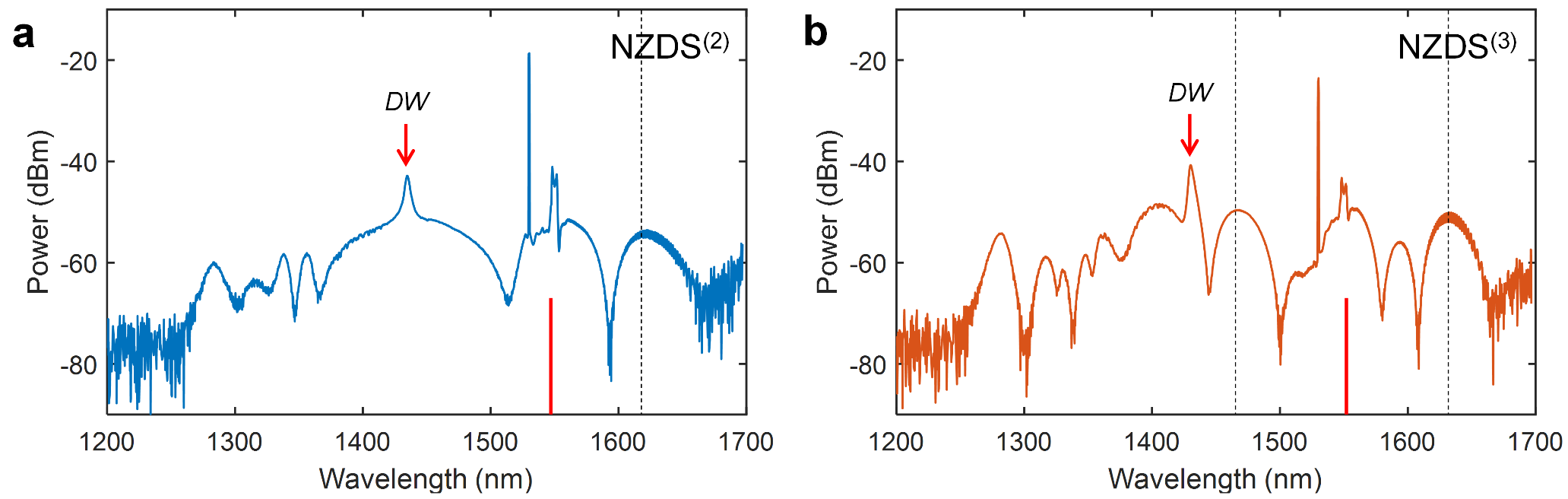


**Fig. S12 Spectral profiles of lower-order NZDS.** The spectrum of **a** NZDS^(2)^ and **b** NZDS^(3)^ with the help of auxiliary laser pump scheme. The black-dashed lines mark the position of prominent combs, and red-solid line marks the position of pump envelopes.

1. Long-term stability of broadband MI microcomb

Compared to the dissipative Kerr soliton state, the MI microcomb state offers a wider accessible range and higher conversion efficiency, and has the potential to be applied in Lidar [S20], communication [S21], and cryptography systems [S22]. Here, we demonstrate a long-term stability experiment in the MI comb state. Since the output power of the F-P microresonator can be used to monitor the state of the optical field, a simple active feedback method can be used to stabilize the optical field. The experimental setup is shown in Fig. S13a. We use 30% of the output power as a feedback signal, and the residual power is sent to an optical spectrum analyzer (OSA) for detection. The wavelength of the laser can be quickly adjusted by the voltage loaded on the piezoelectric ceramic of the laser. The feedback power is locked at −26.5dBm, and its variation is shown in Fig. S13b. The corresponding laser loaded voltage is recorded in Fig. S13c, which also reflects the temperature variation in the laboratory. Fig. S13d exhibits the spectral variation during the experiment, which verifies that the microcomb can operate consistently for more than 15 hours. This device can work for extended periods of time if an automatic bias control circuit is employed to lock the operating point of the intensity modulator (IM).


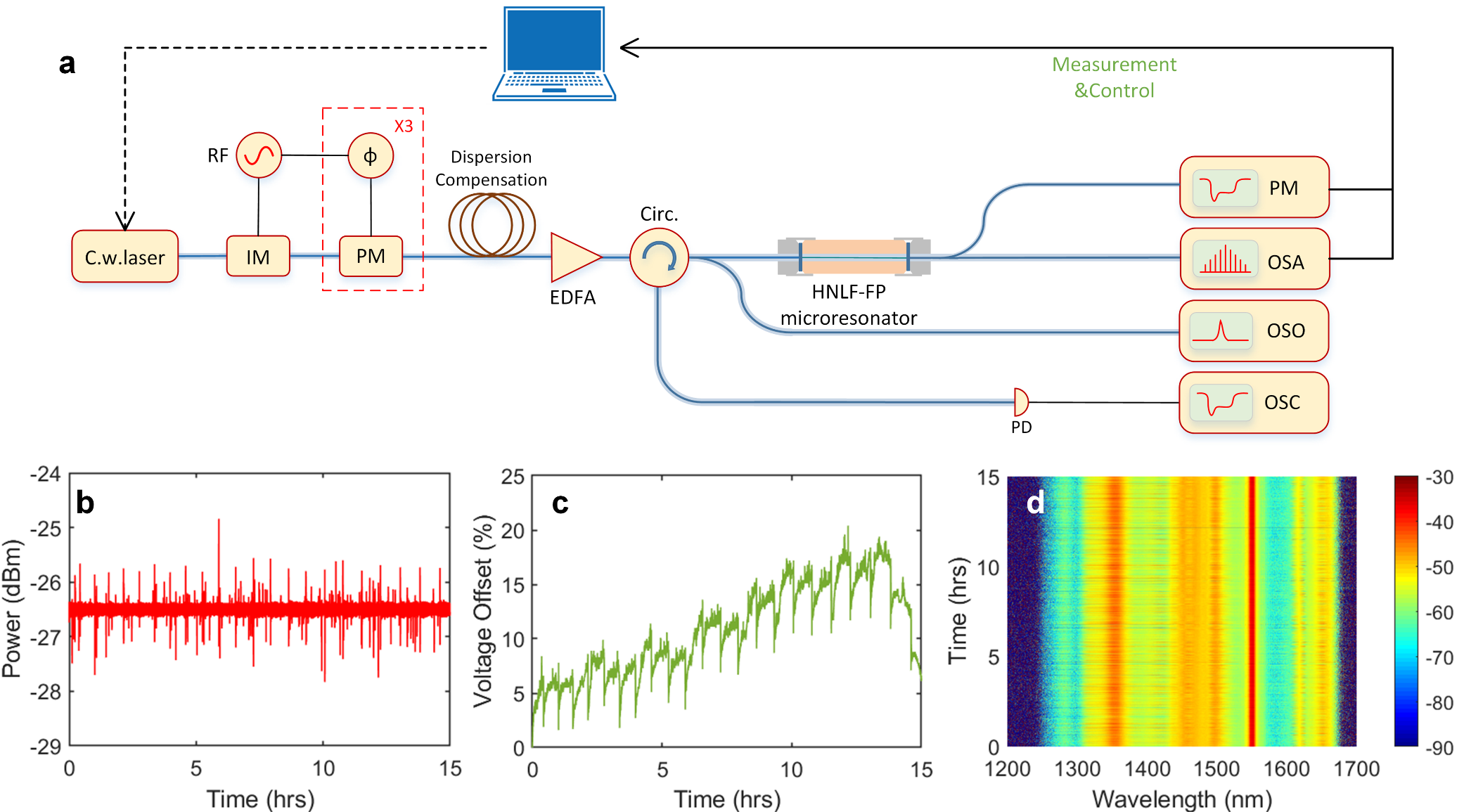


**Fig. S13. Long-term stability experiment of broadband MI microcomb. a** Experimental setup for long-term MI microcomb operation. IM: intensity modulator, PM: phase modulator, EDFA: erbium-doped fiber amplifier, ESA: electronic spectrum analyzer, OSA: optical spectrum analyzer, OSO: optical sampling oscilloscope, OSC: oscilloscope. **b** Power variations of the output microcomb. **c** Voltage applied on piezoelectric ceramic of the laser. **d** Microcomb spectrum variation for 15 hours.

References

1. D. C. Cole, A. Gatti, S. B. Papp, F. Prati, and L. Lugiato, "Theory of Kerr frequency combs in Fabry-Perot resonators," Physical Review A **98**, 013831 (2018).
2. P. Parra-Rivas, D. Gomila, F. Leo, S. Coen, and L. Gelens, "Third-order chromatic dispersion stabilizes Kerr frequency combs," Opt. Lett. **39**, 2971-2974 (2014).
3. G. Genty, S. Coen, and J. M. Dudley, "Fiber supercontinuum sources (Invited)," J. Opt. Soc. Am. B **24**, 1771-1785 (2007).
4. M. Yu, B. Desiatov, Y. Okawachi, A. L. Gaeta, and M. Lončar, "Coherent two-octave-spanning supercontinuum generation in lithium-niobate waveguides," Opt. Lett. **44**, 1222-1225 (2019).
5. V. Brasch, M. Geiselmann, T. Herr, G. Lihachev, M. H. P. Pfeiffer, M. L. Gorodetsky, and T. J. Kippenberg, "Photonic chip–based optical frequency comb using soliton Cherenkov radiation," Science **351**, 357 (2016).
6. J. K. Jang, M. Erkintalo, S. G. Murdoch, and S. Coen, "Observation of dispersive wave emission by temporal cavity solitons," Opt. Lett. **39**, 5503-5506 (2014).
7. P. Parra-Rivas, D. Gomila, and L. Gelens, "Coexistence of stable dark- and bright-soliton Kerr combs in normal-dispersion resonators," Physical Review A **95**, 053863 (2017).
8. I. Hendry, B. Garbin, S. G. Murdoch, S. Coen, and M. Erkintalo, "Impact of desynchronization and drift on soliton-based Kerr frequency combs in the presence of pulsed driving fields," Physical Review A **100**, 023829 (2019).
9. M. H. Anderson, W. Weng, G. Lihachev, A. Tikan, J. Liu, and T. J. Kippenberg, "Zero dispersion Kerr solitons in optical microresonators," Nature Communications **13**, 4764 (2022).
10. M. Karpov, H. Guo, A. Kordts, V. Brasch, M. H. P. Pfeiffer, M. Zervas, M. Geiselmann, and T. J. Kippenberg, "Raman Self-Frequency Shift of Dissipative Kerr Solitons in an Optical Microresonator," Physical Review Letters **116**, 103902 (2016).
11. Y. Okawachi, M. Yu, V. Venkataraman, P. M. Latawiec, A. G. Griffith, M. Lipson, M. Lončar, and A. L. Gaeta, "Competition between Raman and Kerr effects in microresonator comb generation," Opt. Lett. **42**, 2786-2789 (2017).
12. F. Shun and T. Takasumi, "Dispersion engineering and measurement of whispering gallery mode microresonator for Kerr frequency comb generation," Nanophotonics **9**, 1087-1104 (2020).
13. H. Zhou, Y. Geng, W. Cui, S.-W. Huang, Q. Zhou, K. Qiu, and C. Wei Wong, "Soliton bursts and deterministic dissipative Kerr soliton generation in auxiliary-assisted microcavities," Light: Science & Applications **8**, 50 (2019).
14. T. Herr, V. Brasch, J. D. Jost, C. Y. Wang, N. M. Kondratiev, M. L. Gorodetsky, and T. J. Kippenberg, "Temporal solitons in optical microresonators," Nat. Photonics **8**, 145 (2013).
15. H. Guo, M. Karpov, E. Lucas, A. Kordts, M. H. P. Pfeiffer, V. Brasch, G. Lihachev, V. E. Lobanov, M. L. Gorodetsky, and T. J. Kippenberg, "Universal dynamics and deterministic switching of dissipative Kerr solitons in optical microresonators," Nature Physics **13**, 94 (2016).
16. S. Coen and M. Erkintalo, "Universal scaling laws of Kerr frequency combs," Opt. Lett. **38**, 1790-1792 (2013).
17. Q. Li, T. C. Briles, D. A. Westly, T. E. Drake, J. R. Stone, B. R. Ilic, S. A. Diddams, S. B. Papp, and K. Srinivasan, "Stably accessing octave-spanning microresonator frequency combs in the soliton regime," Optica **4**, 193-203 (2017).
18. Y. Xu, A. Sharples, J. Fatome, S. Coen, M. Erkintalo, and S. G. Murdoch, "Frequency comb generation in a pulse-pumped normal dispersion Kerr mini-resonator," Opt. Lett. **46**, 512-515 (2021).
19. 19. S. Malaguti, M. Conforti, and S. Trillo, "Dispersive radiation induced by shock waves in passive resonators," Opt. Lett. **39**, 5626-5629 (2014).
20. 20. A. Lukashchuk, J. Riemensberger, A. Tusnin, J. Liu, and T. Kippenberg, "Chaotic micro-comb based parallel ranging," arXiv:2112.10241 (2021).
21. 21. D. Esman, V. Ataie, B. P. P. Kuo, N. Alic, and S. Radic, "Subnoise Signal Detection and Communication," J. Lightwave Technol. **34**, 5214-5219 (2016).
22. 22. G. Chen, Y. Mao, and C. K. Chui, "A symmetric image encryption scheme based on 3D chaotic cat maps," Chaos, Solitons & Fractals **21**, 749-761 (2004).
